# Silicon flexoelectronic transistors

**DOI:** 10.1126/sciadv.add3310

**Published:** 2023-03-10

**Authors:** Di Guo, Pengwen Guo, Lele Ren, Yuan Yao, Wei Wang, Mengmeng Jia, Yulong Wang, Longfei Wang, Zhong Lin Wang, Junyi Zhai

**Affiliations:** ^1^ CAS Center for Excellence in Nanoscience, Beijing Institute of Nanoenergy and Nanosystems, Chinese Academy of Sciences, Beijing 101400, P. R. China.; ^2^ School of Chemistry and Chemical Engineering, Center on Nanoenergy Research, School of Physical Science and Technology, Guangxi University, Nanning 530004, P. R. China.; ^3^ School of Nanoscience and Technology, University of Chinese Academy of Sciences, Beijing 100049, P. R. China.; ^4^ Georgia Institute of Technology, Atlanta, GA 30332, USA.

## Abstract

It is extraordinarily challenging to implement adaptive and seamless interactions between mechanical triggering and current silicon technology for tunable electronics, human-machine interfaces, and micro/nanoelectromechanical systems. Here, we report Si flexoelectronic transistors (SFTs) that can innovatively convert applied mechanical actuations into electrical control signals and achieve directly electromechanical function. Using the strain gradient–induced flexoelectric polarization field in Si as a “gate,” the metal-semiconductor interfacial Schottky barriers’ heights and the channel width of SFT can be substantially modulated, resulting in tunable electronic transports with specific characteristics. Such SFTs and corresponding perception system can not only create a high strain sensitivity but also identify where the mechanical force is applied. These findings provide an in-depth understanding about the mechanism of interface gating and channel width gating in flexoelectronics and develop highly sensitive silicon-based strain sensors, which has great potential to construct the next-generation silicon electromechanical nanodevices and nanosystems.

## Teaser

Si flexoelectronic transistors and corresponding perception system for Si-based electromechanical interaction is developed.

## INTRODUCTION

Silicon (Si) is an internationally proverbial underlying semiconductor of conventional field-effect transistors (FETs) that retains as an inevitable indispensable electronic component (*1*). The well-developed Si technology not only conveniently facilitates integration with electrodes, dielectrics, and other elements to form elements of electronic circuit transistors (*2*) but also provides the serviceability for large-scale integration, which has great applications in energy harvesting (*3*), electronics (*4*), and sensing (*5*). The Si-based electronic device controlled by external gate voltage can be used as human-machine components for information monitoring and intelligent identification through the mechanical strain-induced passive electrical signal change (*6*, *7*). However, it is extraordinarily difficult to interface the human/ambient mechanical signals directly with the current Si-based technology (*8*–*10*), which is not only limited by the intricate integration of heterogeneous components at small size (*11*) but also confined by the lack of active interaction between electronic components and mechanical action. Si does not have piezoelectric property so that it is insensitive to mechanical triggering.

Piezotronics were invented to accomplish the immediate interrelation between biomechanical motion and electronic devices of the third-generation semiconductors, which is analogous to physiological mechanoreceptors in electronic systems (*12*). Using a completely different principle, the piezotronic devices use strain-induced inner crystal piezopotential at the interface/junction as the gate voltage to control the electronic transport process (*13*), which have no requirements on the additional electrical stimulation. The piezotronics has been demonstrated in wurtzite-structured semiconductors, especially in the third-generation semiconductors (ZnO, GaN, SiC, etc.) (*14*–*16*) and the two-dimensional transition metal dichalcogenides (MoS_2_, WS_2_, etc.) (*17*, *18*), which has made important applications in active electronics/optoelectronics (*19*–*21*). By integrating the third-generation semiconductors on Si (*22*–*24*), adaptive sensing in Si-based heterojunction devices is enabled. However, it still rely the mechanism of piezopotential gating in the third-generation semiconductor. The electromechanical interactions with functionalization have not yet been realized directly on Si.

Flexoelectricity is an electromechanical property that reflects on coupling between electrical polarization and a strain gradient that enables mechanical manipulation of polarization (*25*, *26*). The strain gradient can break any material’s inversion symmetry, resulting in a polarization with a preferred direction, inducing “piezoelectric” property in a non-piezoelectric material (*27*–*30*). Recent researches observe the giant flexoelectric effect in centrosymmetric semiconductors (*31*), which has inspired some electronic/optoelectronic phenomena in non-piezoelectric semiconductors, such as flexoelectronics (*32*), flexo-photovoltaic effect (*33*), and photoflexoelectric effect (*34*). It enables the possibility of direct interaction with functionalization between mechanical signals and Si-based electronics through the flexoelectric polarization engineering. In our previous work, by coupling the flexoelectric effect and semiconducting property of centrosymmetric semiconductors, flexoelectronics (or flexotronics) is demonstrated by atomic force microscopy (*32*), which largely expends the piezotronics from the third-generation semiconductor to the conventional semiconductors. The flexoelectronic effect can be used to mechanically switch the electronics in the nanoscale with fast response and high resolution. Nevertheless, the previous research is only a preliminary exploration of flexoelectronics based on AFM. Thus, in-depth understanding the mechanism of the flexoelectronics is important and necessary, and whether it can be used for high-performance devices at macroscopic scale and systems is a matter of great concern, because the realization of high-performance Si-based devices and systems at the macroscopic scale is of great significance for human/ambient-machine interaction. Meanwhile, it can also provide scientific and technical support for the development of flexoelectronics.

Here, we report the first study of Si flexoelectronic transistors (SFTs) and system, which demonstrate that direct interactions between mechanical action and current Si-based technology can be achieved. The strain gradient–induced flexoelectric polarization in Si can effectually regulate the electronic transport characteristics of SFT, including two modulation mechanisms of channel width gating and (dual) interfacial barrier gating. The SFT and the corresponding perception system can enable tactile sensing for the recognition of force location and show high sensitivity with a gauge factor of ~2189, which is more than 10 times higher than that of the state-of-the-art Si nanowire strain sensor (~200) and even much larger than the most of piezoresistive/piezoelectric nanodevices (<2000) (*35*, *36*). This work not only achieves the flexoelectronic transistors and system for direct electromechanical interactions in Si-based electronics but also presents in-depth understandings about the flexoelectronics in both channel width gating and (dual) interfacial barrier gating, which fundamentally pave a way for realizing Si-based electromechanical nanodevices and nanosystems. In addition, it could potentially stimulate the exploration of phenomena in centrosymmetric semiconductors via flexoelectric polarization engineering.

## RESULTS

### Prototype and working mechanism of the flexoelectronic transistors

**Fig. 1.**
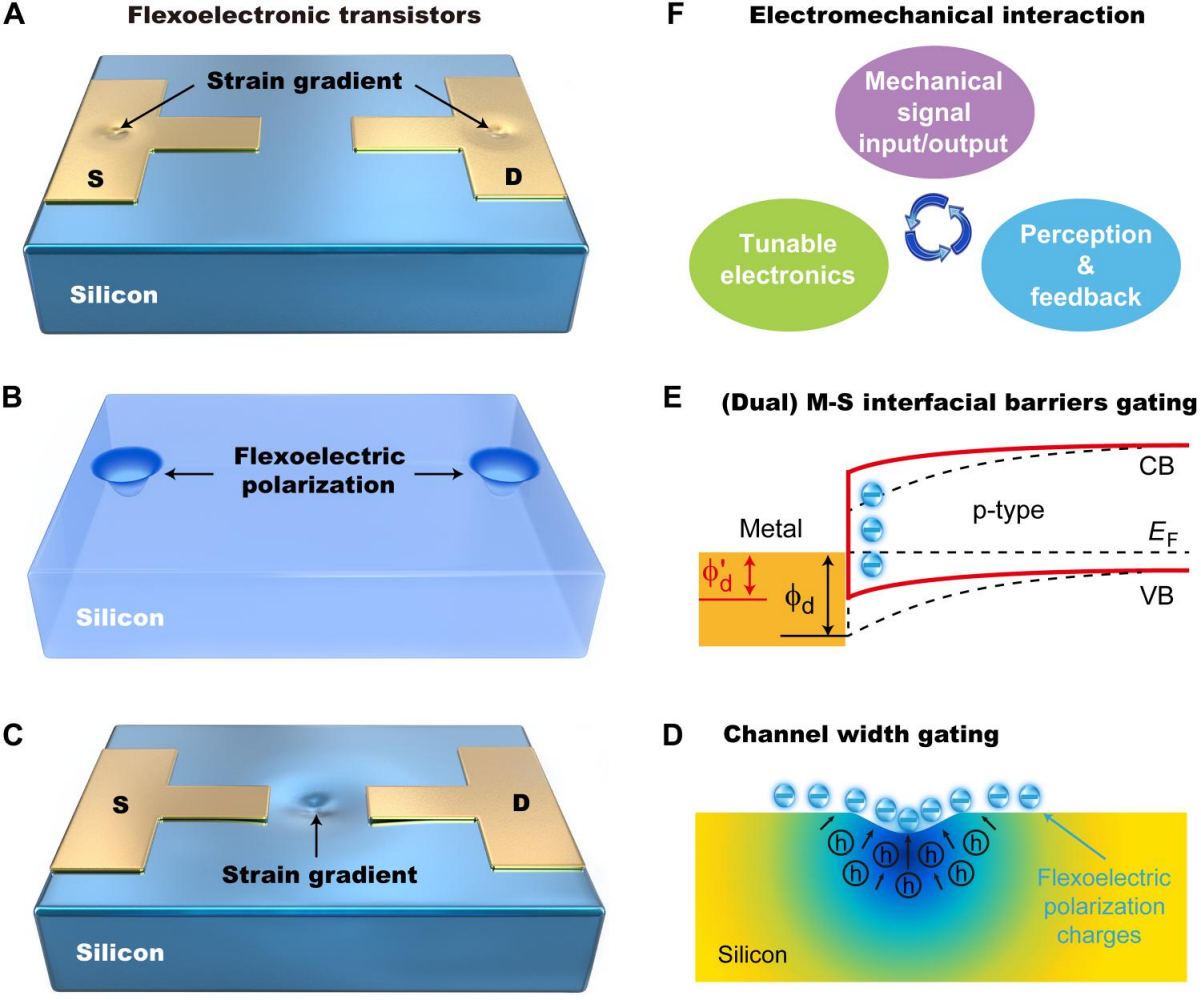
The prototype and mechanism of SFTs. (**A** and **C**) Schematics illustrating the concept of strain gradient gated flexoelectronic transistors proposed here. (**B**) The flexoelectric polarization generated in the inhomogeneous strained crystal, which exerts a substantial influence on the concentration and distribution of free carriers, resulting in the mechanism of (**D**) channel width gating and (**E**) (dual) interfacial barriers gating, (**F**) achieving tunable electronics with specific characteristics for electromechanical interaction. ϕ*_d_* and ϕ*′_d_* represent the interfacial barrier height formed at metal/p-Si contact without (black dashed line) and with (red solid line) strain gradient, respectively. *CB*, *E*_F_, and *VB* represent the conduction band, Fermi surface, and valence band, respectively.

The prototype of SFT is schematically illustrated in Fig. 1 (A to C). Different from the working mechanism of external voltage gating of conventional complementary metal-oxide semiconductor device, the SFTs operate through gating effect of the strain gradient–induced flexoelectric polarization charges (*32*). When applied an inhomogeneous strain on the Si, the flexoelectric polarization field can be created inside the crystal over a range of volumes, which have a substantial effect on the concentration and distribution of free carriers at channel and metal-semiconductor interfaces (Fig. 1, B and C, and fig. S1), resulting in two modulation mechanisms of channel width gating (Fig. 1D) and metal-semiconductor (dual) interfacial barrier gating (Fig. 1E). The magnitude and polarity of the flexoelectric polarization charges within Si depend on the magnitude and polarity of the applied inhomogeneous strain, respectively. Hence, not only the charge carrier transport can be regulated by the strain gradient–induced flexoelectric polarization but also the characteristics of the electric transport can be controlled. The flexoelectronic transistors are a type of device, which uses inner polarization charges of non-piezoelectric Si to directly generate digital signals to control the channel width and the interfacial barrier heights. Therefore, direct interactions with functionalization between mechanical action and Si-based electronics can be realized (Fig. 1F).

### Strain gradient–induced flexoelectric polarization in Si

To demonstrate the working mechanism of channel width gating and interfacial barrier gating of the SFT, we explored the carrier transport properties of SFT by applying inhomogeneous stress on the channel and the metal-Si interfaces, respectively. The external inhomogeneous stress was exerted using a tungsten probe (fig. S2), of which the Young’s modulus (411 GPa) is much higher than that of Si (131 GPa). Thus, the distortion will occur on the Si surface, resulting in a strain gradient and inducing flexoelectric polarization within Si. To obtain the spatial distribution of strain gradient induced by the probe, theoretical investigations of Hertzian contact mechanics (*37*) were performed (text S1 and fig. S1). During the simulation, the tungsten probe is reasonably assumed to be sufficient rigidity without deformation, and the contact area is considered equivalent to the area of probe tip. Figure S1B shows the strain distribution in both the *x* and *z* components of Si under a force of 25 mN with a contact radius of 1 μm, which indicates that the indentation in the contact area can induce effective inhomogeneous gradient distribution in the lateral and vertical direction. The distribution of the corresponding flexoelectric polarization field is shown in fig. S1C, and the theoretical calculation (*32*, *38*–*40*) is detailed in text S2. The out-of-plane polarization *P* distributes in the form of a nonuniform semielliptical diffusion and reaches a maximum of ~10^−4^ C m^−2^ beneath the center of the contact region. The flexoelectric polarization generated in the experiments may be orders of magnitude higher than the theoretical result. The primary ratiocination for this enhancement may be due to that the effective flexoelectric coefficient was theoretically calculated at 0 K, while the experimental results were carried out at room temperature and intensively relied on the intrinsic static permittivity, defect, or doping state of the material (*38*, *39*). Consequently, these results demonstrate that large strain gradient and effective flexoelectric polarization can be created and induced in Si by the micrometer-scale probe indentation.

### Channel width gating of the flexoelectronic transistor

**Fig. 2.**
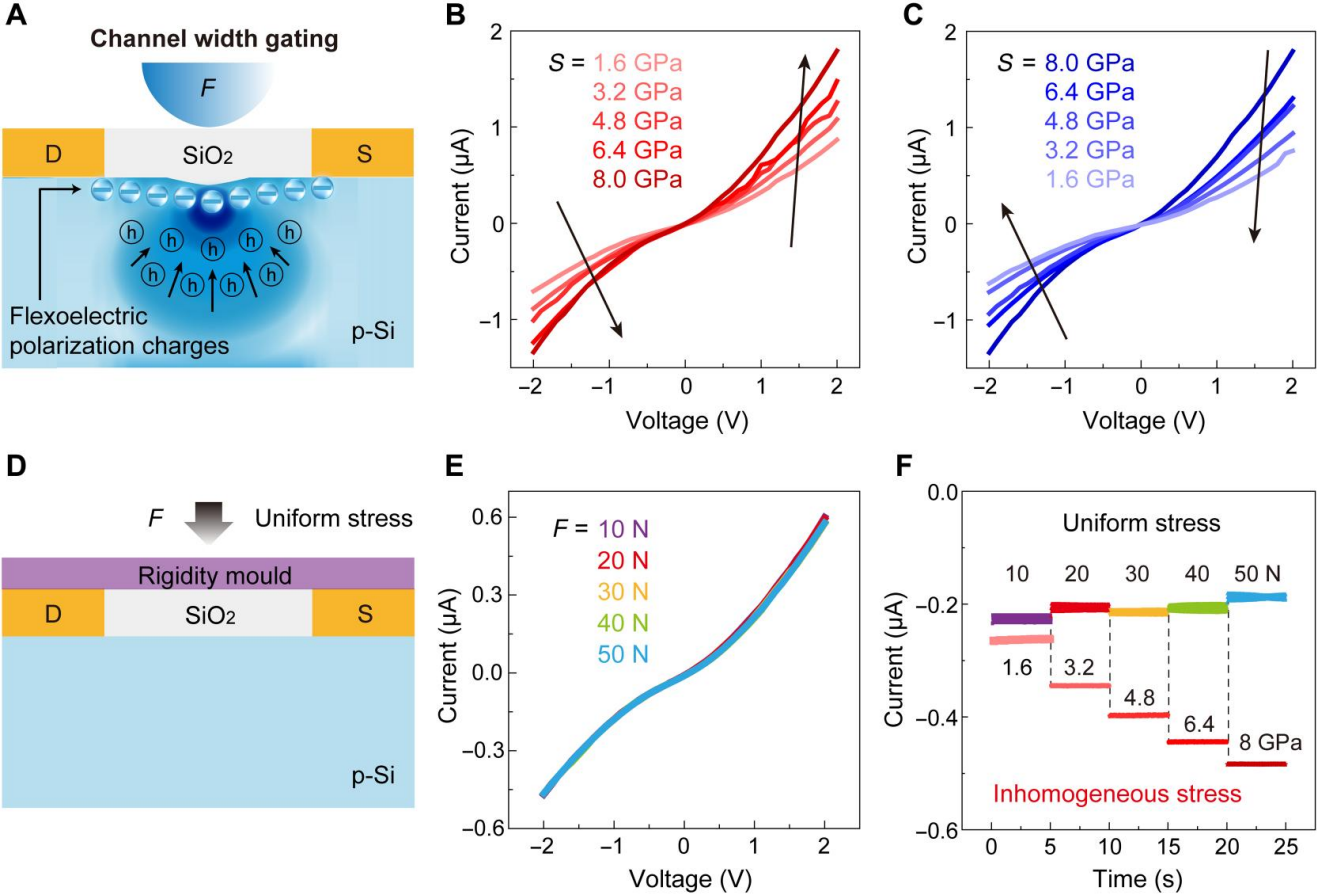
Channel width gating effect of the SFT. (**A**) Schematic illustrating the channel width gating effect of SFT. Strain gradient–induced negative flexoelectric polarization charges attract holes and form an enhanced region at channel. (**B** and **C**) The symmetric modulation of electrical transport of the device under nonuniform stress. (**D**) Schematic illustrating the Si transistor under uniform stress. (**E**) The negligible modulation of electrical transport under uniform stress. (**F**) The current response of Si transistors under loading stress [inhomogeneous stress shown in (A) and uniform stress shown in (D)] with a step-by-step increase at a fixed bias of −1 V.

We first fabricated FETs based on commercial p-type silicon wafer and characterized their performance at room temperature (figs. S3 to S5). The Si FETs designed with a three-terminal structure consist of gold-plated electrodes at source/drain and a 5-μm-wide channel. To elaborate the channel width gating effect by the strain gradient–induced flexoelectric polarization, we characterized the electrical transport properties of a Si FET under an indentation on the channel. For the flexoelectronic transistor, the current-voltage relation (*I*-*V*) characteristics show a near-ohmic behavior and are highly symmetric. The forward and reverse currents are both increasing steadily with the increase of loading forces (Fig. 2B), while the opposite trend of current changes is observed when gradually relieved the stress (Fig. 2C). It can be seen that, even with a small force (5 mN), a large current change occurs. The dynamic current variations with increasing loading forces at a fixed location on the channel are consistent with the above results of electrical measurements under static forces, further demonstrating the effectiveness of the gating effect of applied stress on the channel (Fig. 2F). Same electrical characterizations of other devices have also been carried out, which indicates the reproducibility and repeatability of the mechanically tunable electronics (figs. S6 and S7).

In general, the piezoresistive effect will be induced when applied a loading force, whereas it cannot explain the above anomalous behavior. Previous studies show that the piezoresistive effect in bulk Si is much smaller, which has also been demonstrated by applying uniform forces on the Si FET (Fig. 2, D to F). The current has an inappreciable variation with gradually increased loading forces. On the other hand, the piezoresistive coefficient of p-type Si is a negative value in the same configuration with this work (fig. S8 and text S3), which is in contrast to our observations. The contingency caused by artificial or other factors has also been excluded through a series of comparative experiments (fig. S9).

The predominant role of the mechanically tunable Si-based electronics is the strain gradient–induced flexoelectric polarization at the channel. Figure 2A shows the proposed mechanism of the channel width gating effect of the flexoelectronic transistor. Under probe indentation, the strain gradient will be created at the inner oxide layer and Si, resulting in the effective flexoelectric polarization charges presented at the interface of channel region. The negative flexoelectric polarization charges attract the holes along the polarization field direction, which results in the accumulation of holes and the formation of an enhanced region near the top interface of channel, and increase the width of the effective conductive region of the channel (fig. S11). Consequently, the electrical transports of the flexoelectronic transistors are substantially enhanced by the channel width gating effect. Theoretical simulations further reveal that the inner flexoelectric polarization can effectively modulate the distribution and concentration of holes near the interface (fig. S10).

### Interfacial barrier gating of the flexoelectronic transistor

**Fig. 3.**
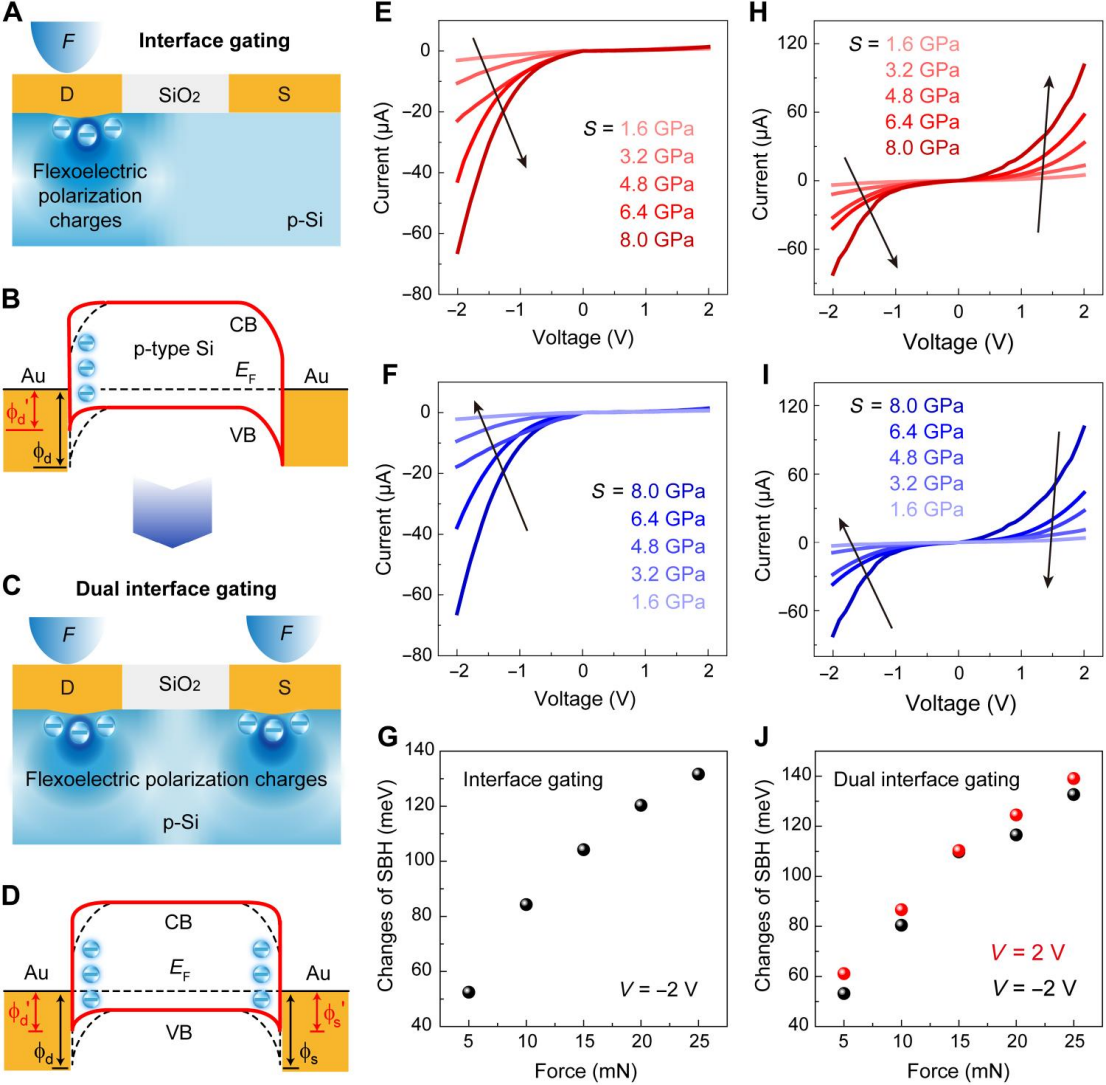
(Dual) interfacial barrier gating effect of SFT. (**A**) Schematic illustration of the SFT with an indentation applied on the drain. The blue negative charges represent flexoelectric polarization charges, which are immobile. (**B**) Band diagrams showing the mechanism of the interfacial barrier gating. The black dashed line and the red solid line represent the energy band profiles without and with the flexoelectric polarization charges, respectively. ϕ*_d_* and ϕ*′_d_* represent the interfacial barrier height formed at metal/p-Si contact, respectively. *CB*, *E*_F_, and *VB* represent the conduction band, Fermi surface, and valence band, respectively. (**C**) Schematic illustration of the SFT with indentations applied on both of drain and source. The blue negative charges represent flexoelectric polarization charges, which are immobile. (**D**) Band diagrams showing the mechanism of the dual interfacial barriers’ gating. ϕ*_d_* and ϕ*′_d_* as well as ϕ*_s_* andϕ*′_s_* represent the interfacial barriers’ heights formed at metal/p-Si contacts, respectively. (**E** and **F**) The asymmetric modulation of electrical transport with Schottky characteristics by interfacial barrier gating. (**G**) Variation of SBH derived from the *I*-*V* curves of (E). (**H** and **I**) The symmetric modulation of electrical transport with Schottky characteristics by dual interfacial barriers’ gating. (**J**) Variation of SBH derived from the *I*-*V* curves of (H).

To explore the modulation effect of the flexoelectric polarization on the interfacial barrier, we investigated the *I*-*V* characteristics of the Si FET by applying mechanical loading force at the metal-semiconductor interface (Fig. 3A). With the loading force increased from 5 to 25 mN, the reverse current of the SFT increases notably, whereas the forward current was almost unchanged. The *I*-*V* curve gradually changes to a strict Schottky characteristic, which exhibits a rectification behavior and become highly asymmetric (Fig. 3E). This regulation is reversible when the force is removed (Fig. 3F). The static repeatable measurements of other devices have been carried out, illustrating the reproducibility of the modulation process (figs. S12 and S13). These results demonstrate that the mechanical action can modulate not only the magnitude of the charge carrier transport of the Si transistor but also its characteristics.

Figure 3B illustrates the mechanism of the interface gating effect of SFT. When applied a probe force on the drain, the strain gradient induces the negative flexoelectric polarization charges and hence the negative flexopotential presented near the Au-Si interface, which can attract the holes near the interface and result in a less depleted region. Thus, it can substantially lower the interfacial Schottky barrier and hence increase the electrical transport across the Au-Si contact at drain. The detailed evolution of interfacial barrier height of the Au-Si contact under probe indentation is shown in fig. S14. Nevertheless, the interfacial barrier at source under reversely biased is still large enough to limit the charge carrier transport. The effective changes of SBH of the flexoelectronic transistor under various loading forces were derived from the *I*-*V* curves using the classic Schottky theory (Fig. 3G) (*13*). The effective changes of SBH (Δϕ) can be estimated by the following formula: Δϕ *= −kT* ln(*I*_strain_/*I*_free_). Effective change of SBH can be found with the increase of loading force from 5 to 25 mN and reaches the maximum of 131.6 meV at 25 mN, which is similar to the scenario of the Schottky barrier modulation of the piezotronic theory (*12*, *13*) and further confirming the effectiveness of interfacial barrier gating effect by strain gradient–induced flexoelectric polarization.

### Dual interfacial barrier gating of the flexoelectronic transistor

To in-depth understand the physics of the interface gating effect of SFT, we performed the same experiments, but applying loading forces on the interfaces of source and drain at the same time (Fig. 3C). In this case, negative flexoelectric polarization charges will be generated at both of the Au-Si interfaces, which can simultaneously lower both interfacial barriers of the metal-semiconductor contacts and hence notably enhance the electrical transport of SFT (Fig. 3D and fig. S15). Therefore, the charge carrier transport across both interfacial barriers can be controlled effectively by the strain gradient–induced negative flexoelectric potential. On the basis of the fundamental principle, an enhanced SFT is introduced to gigantically modify the electronic transport property by designing the dual interfacial barriers’ synergistic modulation.

The electrical transport properties of SFT under various loading forces were characterized. As expected, when increasing the force from 5 to 25 mN, both of the forward current and reverse current are synchronously increased (Fig. 3H and figs. S16 and S17). The Schottky curves exhibit a symmetrical change, which is much different from the cases of channel width gating and the single interfacial barrier gating. Besides, the electrical transport characteristics of SFT can restore to its initial state with the decrease of the loading forces (Fig. 3I). The effective change of interfacial SBH as a function of loading force was obtained according to the classical Schottky theory. The result shows that the SBHs substantially decrease with increasing the loading force, and the magnitude of the changes are similar at forward and reverse voltages (Fig. 3J). The maximum variation of the Schottky barrier reaches 140 meV at a force of 25 mN, which is visibly larger than that of the interfacial gating on one side. The consistency of the proposed principle, experimental results, and theoretical analysis powerfully demonstrates the dual interface gating effect of the SFT, indicating its great potential for direct electromechanical interactions. In addition, it should be pointed out that the mechanisms of channel width gating effect and (dual) interfacial barrier gating effect of SFTs are universal and can be applicable to any type of semiconductors, further extending the piezotronics and ferroelectrics of piezoelectric/ferroelectric semiconductors to centrosymmetric semiconductors.

### Sensing performance of the flexoelectronic transistors

**Fig. 4.**
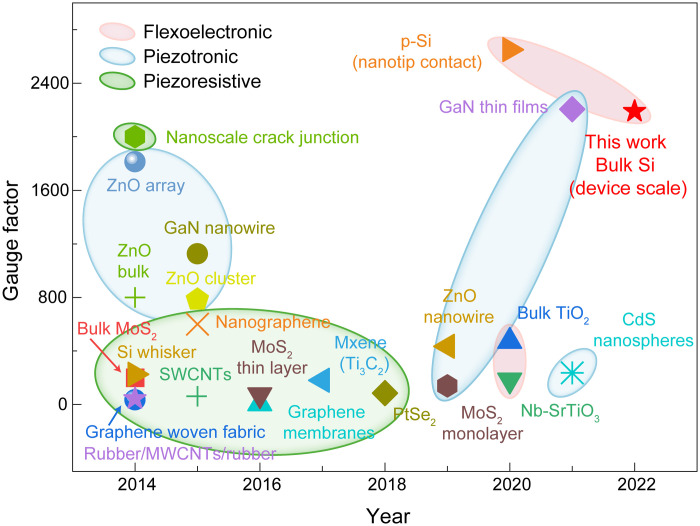
Sensing performance of the SFTs. Comparison of the gauge factor of flexoelectronic transistors with some existing piezoresistive/piezotronic-based strain sensors. SWCNTs, single-walled carbon nanotubes; MWCNTs, multiwalled carbon nanotubes.

To preliminarily estimate the sensitivity of SFT, we characterized the gauge factor (*41*), a dominant indicator of the strain sensing, which is defined as the ratio of relative change in current or resistance to the mechanical strain (text S4). The corresponding values of SFTs under various strains in channel width gating and (dual) interface gating were obtained (fig. S18). The gauge factors of all three gating modes increase notably with the increase of the strain, which shows the effectiveness of the “gating” effect of flexoelectric polarization within the channel or at the metal-semiconductor interfaces. Compared the value of 76 in the channel width gating, the gauge factor of the dual interfacial barriers’ gating has orders of magnitude improvement and reaches 2189, indicating that the sensitivity of interface gating is much higher than the channel width gating of SFT. The results are consistent with the previous piezotronic effect that uses piezoelectric polarization of piezoelectric materials, of which the interface gating is more effective in these cases. The strain sensitivity of SFT is more than 10 times higher than that of the state-of-the-art Si nanowire–based strain sensor (~200) and even larger than the most of piezoresistive/piezoelectric nanodevices (<2000) (Fig. 4 and table S1) (*17*, *42*–*60*). The sensitivity of SFT could be further improved once the dimension (including the channel length) of Si reduces to nanometer scale. In this configuration, the flexoelectric polarization can be induced throughout the crystal, which has a huge impact on the concentration and distribution of free carriers in all regions of the crystal. These results indicate that the strain gradient is a simple but effective route for the control of electrical transport of Si-based device, showing the advantages of flexoelectronic transistor for highly sensitive strain sensing.

### A prototype of perception system based on flexoelectronic transistors and the convolutional neural networks for mechanical signal identification

The mechanical tunable electronics of SFT show their own specific characteristics under different gating modes, respectively (Figs. 2 and 3). In channel width gating mode, the electrical transport curves show a near-ohmic behavior and symmetric change. The *I*-*V* curves gradually change to strict Schottky characteristics, exhibiting a rectification behavior in interfacial barrier gating mode, whereas presenting a symmetrical change in dual interfacial barriers’ gating. Thus, the flexoelectronic transistor can not only detect the magnitude of the loading force but also identify the location where the force is applied in view of the specific characteristics of electrical transport. On the basis of the above results, a prototype of the perception system based on SFTs is developed, the overview and process of which are shown in fig. S19. The proposed system consists of two processes, the mechanosensory process and identification process. First, the model training process was performed using a translation stage with a predefined probe stress to touch different locations of SFT. The generated electronic signals were collected and divided into four categories of “I,” “D,” “S,” and “DS.” Thus, the relationships between mechanical action and electronic signal are established. During the mechanosensory process, once the mechanical actions interface with perceptual component, the touch signals can be converted into electronic signals, which is similar to the process of mechanosensation in physiology.

To further evaluate the accuracy of the mechanical signal identification, we constructed the convolutional neural network (CNN) model, an effective tool widely used in deep learning. The dataset (experimental *I-V* curves under different gating modes) obtained was input into the CNN model, and the feature extraction and data dimension reduction were performed by the convolutional layers and max-pooling layers (fig. S20). With the help of the CNN model with clustering performance, it is more convenient to qualitatively extract the features under various touch categories and eventually achieve high-precision recognition of the mechanical action signals in identification process. The recognition accuracy and cross-entropy loss of mechanical signal identification with different training epochs were characterized. As the training epoch increases, the identification accuracy increases substantially. After the training process in the CNN model with 50 training epochs, 96.95% of the predicted labels are matched the authentic labels in the validation process, showing a high recognition accuracy of mechanical signals. This capability of perception system may enable the Si-based technology to realize human-machine interface with enhanced security.

## DISCUSSION

In summary, we developed the SFTs and achieved direct interactions with functionalization between the mechanical action and the Si-based electronics using the inner crystal flexoelectric polarization field created at channel and metal-semiconductor interfaces under strain gradient to substantially modulate charge carrier transport. The mechanical tunable electronics with specific characteristics arise largely from two effects: the channel width gating effect and the (dual) interfacial barrier gating effect. The flexoelectronic transistor–based perception system exhibits capability to identify the location of external mechanical forces with high strain sensitivity. This study opens up the possibility of realizing Si-based electromechanical interfaces, enriches the understanding of flexoelectronics, and can also inspire physics in centrosymmetric semiconductors via flexoelectric polarization engineering.

## MATERIALS AND METHODS

### Fabrication of Si transistors

The fabrication process of the Si transistors is schematically illustrated in fig. S3. First, a silicon oxide wafer with a sandwich structure was pretreated sequentially with acetone, ethanol, and deionized water. The silicon oxide wafer has a top 270-nm-thick SiO_2_, a middle 500-μm-thick p-type (100)-oriented Si (resistivity, 1 to 3 ohm cm), and a bottom 270-nm thick SiO_2_. Then, part of the top SiO_2_ surface was masked by photolithography (M1) (SUSS MA/BA 6) using a negative photoresist SUN-9i, followed by inductively coupled plasma (SI500) etching to open the electrode areas and immersing the wafer in hot acetone to remove the photoresist layer. After cleaning, the top SiO_2_ layer was patterned again through lithography (M1) using a negative photoresist SUN-9i, and Cr/Au (10/40 nm) metals were deposited on the surface by radiofrequency magnetron sputtering (Denton Discovery 635), and then the source and drain electrodes were fabricated by a conventional liftoff process. In addition, the top surface layer was patterned by lithography (M2), and then Cr/Au (10/40 nm) electrodes were deposited followed by the parallel liftoff process to extend the source and drain electrodes. Last, a Cr/Au (10/40 nm) metal layer was deposited in the same way on the bottom surface as the bottom gate electrode, and the organic contaminants were removed by thermal annealing at 350°C for 5 hours.

### Characterizations of Si transistors

The cross-sectional morphology and thickness of a Si transistor were characterized using a scanning electron microscope (SU8020, Hitachi). A top-view optical micrograph in fig. S4A shows the successful fabrication of a Si transistor with a channel width of 5 μm. The cross-sectional structures at the electrode (mark 1) and the channel (mark 2) were characterized as shown in fig. S4B and the ~270-nm SiO_2_/Si interface as shown in fig. S4C, compared with the pristine 270-nm SiO_2_/Si of the silicon oxide wafer in fig. S4D, which also proves that the SiO_2_ on top of the channel was accurately retained during etching. In fig. S4 (E and F), the cross-sectional characterization of the metal-oxide semiconductor demonstrates the successful extraction of the top gold electrode. The electrical characteristics of Si transistors were measured using a semiconducting parameter analyzer (Keithley 4200). The translation stage with conductive tungsten probes (radius, 1 μm) was used to apply compressive forces and simultaneously measure the current. In the dual interfacial barrier gating model, a homemade system was used to apply the forces by integrating a high-precision dynamometer and translation probe stage. The electronic devices were first placed on a high-precision dynamometer and then used the precision translation stage with conductive tungsten probes to apply compressive forces on the drain and source. The static force can be precisely controlled by the stage and read out using the dynamometer.
