# Supplementary material for: Silicon flexoelectronic transistors
Source: Sci Adv. 2023 Mar 10;9(10):eadd3310. doi: 10.1126/sciadv.add3310 (PMC10005167; doi:10.1126/sciadv.add3310)
Supplement: Supplementary file 1 — Text S1 to S4 Figs. S1 to S20 Table S1 [file sciadv.add3310_sm.pdf]

Science Advances
AAAS

Supplementary Materials for

Silicon flexoelectronic transistors

Di Guo *et al.*

Corresponding author: Longfei Wang, lfwang12@binn.cas.cn; Zhong Lin Wang, zhong.wang@mse.gatech.edu; Junyi Zhai, jyzhai@binn.cas.cn

Sci. Adv. **9**, eadd3310 (2023)
DOI: 10.1126/sciadv.add3310

This PDF file includes:

Text S1 to S4
Figs. S1 to S20
Table S1

Supplementary Text 1

Theoretical calculation of strain distribution in Si

In this work, Hertz contact mechanics(*37*) is used to calculate analytically spatial distributions of strain and strain-gradient in the cylindrical coordinate space with the center of the contact area as the origin under a spherical indenter. The superposition principle with Boussinesq and Cerruti gives the strain and strain gradient distribution under a mechanically loaded probe-force. Here, the normal indentation distribution function of the r and z directly beneath the indenter can be described as：

$$\frac{\sigma_z(r)}{p_{\mathrm{m}}} = -\frac{3}{2}\left(1-\frac{r^2}{a^2}\right)^{1/2} \quad r \le a \tag{1}$$

Where σ_z, P_{m} and a represent a z-component of normal stress, a mean pressure of the entire contact area under the load force and a contact radius, respectively. Meanwhile, the contact depth and radius can be expressed as:

$$h = \left(\frac{3F}{4E\sqrt{R}}\right)^{2/3} \tag{2}$$

$$\mathrm{a} = \left(Rh\right)^{1/2} \tag{3}$$

where h represents the indentation depth, F represents the pressure, E is called the elastic modulus or Young's modulus, and R denotes the radius of the spherical indenter. By solving the basic equation of displacement method for spatial axisymmetric problems, the calculation formulas of stress and displacement components can be obtained as follows:

$$\sigma_x = \frac{F_0}{2\pi}\left[\frac{1-2\nu}{r^2}\left(\left(1-\frac{z}{\rho}\right)\frac{x^2-y^2}{r^2}+\frac{zy^2}{\rho^3}\right)-\frac{3zx^2}{\rho^5}\right] \tag{4}$$

$$\sigma_y = \frac{F_0}{2\pi}\left[\frac{1-2\nu}{r^2}\left(\left(1-\frac{z}{\rho}\right)\frac{y^2-x^2}{r^2}+\frac{zx^2}{\rho^3}\right)-\frac{3zy^2}{\rho^5}\right] \tag{5}$$

$$\sigma_z = -\frac{3F_0}{2\pi}\frac{z^3}{\rho^5} \tag{6}$$

$$\tau_{xy} = \frac{F_0}{2\pi}\left[\frac{1-2\nu}{r^2}\left(\left(1-\frac{z}{\rho}\right)\frac{xy}{r^2}-\frac{xyz}{\rho^3}\right)-\frac{3xyz}{\rho^5}\right] \tag{7}$$

$$\tau_{xz} = -\frac{3F_0}{2\pi}\frac{xz^2}{\rho^5} \tag{8}$$

$$\tau_{yz} = -\frac{3F_0}{2\pi}\frac{yz^2}{\rho^5} \tag{9}$$

where σ_x, σ_y, σ_z are the stress of the x, y, z component, respectively. F_0 represents the point force at the origin, and ν represents the Poisson's ratio. r, z are the two coordinates of the observed point, $r^2 = x^2 + y^2$. ρ is the distance from the observed point to the origin of the coordinate, $\rho = (r^2 + z^2)^{1/2}$. With increasing of ρ, the stress and displacement components decrease noticeably. When $\rho \to \infty$, both the stress and displacement components approximately tend to zero, indicating that the stress and displacement of the object under the loading force state have local properties. With superposition principle, the strain distribution under a locally loading tip-force can be obtained as:

$$\frac{\sigma_r}{p_\mathrm{m}} = \frac{3}{2}\left\{\frac{1-2\nu}{3}\frac{a^2}{r^2}\left[1-\left(\frac{z}{u^{1/2}}\right)^3\right]+\left(\frac{z}{u^{1/2}}\right)^3\frac{a^2u}{u^2+a^2z^2}\right.$$
$$\left.+\frac{z}{u^{1/2}}\left[\frac{u(1-\nu)}{a^2+u}+(1+\nu)\frac{u^{1/2}}{a}\tan^{-1}\left(\frac{a}{u^{1/2}}\right)-2\right]\right\} \tag{10}$$

$$\frac{\sigma_\theta}{p_\mathrm{m}} = -\frac{3}{2}\left\{\frac{1-2\nu}{3}\frac{a^2}{r^2}\left[1-\left(\frac{z}{u^{1/2}}\right)^3\right]+\frac{z}{u^{1/2}}\left[2\nu+\frac{u(1-\nu)}{a^2+u}-(1+\nu)\frac{u^{1/2}}{a}\tan^{-1}\left(\frac{a}{u^{1/2}}\right)\right]\right\} \tag{11}$$

$$\frac{\sigma_z}{p_\mathrm{m}} = -\frac{3}{2}\left(\frac{z}{u^{1/2}}\right)^3\left(\frac{a^2u}{u^2+a^2z^2}\right) \tag{12}$$

$$\frac{\tau_{rz}}{p_\mathrm{m}} = -\frac{3}{2}\left(\frac{rz^2}{u^2+a^2z^2}\right)\left(\frac{a^2u^{1/2}}{u+a^2}\right) \tag{13}$$

$$u=\frac{1}{2}\left\{(r^2+z^2-a^2)+\left[(x^2+y^2-a^2)^2+4a^2z^2\right]^{1/2}\right\} \tag{14}$$

Where σ_r represents radial stress. According to the generalized Hooke's law, considering the strains of stresses σ_i, σ_j, σ_k acting simultaneously in the i-axis direction, the strain ε distribution will be:

$$\varepsilon_i=\frac{1}{E}\left[\sigma_i-\nu(\sigma_j+\sigma_k)\right] \tag{15}$$

$$\varepsilon_{ij}=\frac{1+\nu}{E}\tau_{ij} \tag{16}$$

Each independent strain gradient component can be calculated by taking partial derivatives of i, j or k. In the theoretical simulation, the Young's modulus of Si is 131 Gpa, the Poisson's ratio is 0.2782, the tip radius is 1 μm, and the loaded tip force is 25 mN. Consequently, a local non-uniform gradient variation spatial distribution from the contact center point to the three-dimensional diffusion is displayed

at different force positions involved in three gating modes of channel-width gating, interfacial barrier gating and dual interfacial barriers' gating, respectively (**fig. S2**), which presents a three-dimensional semi-ellipsoid distribution as a whole. In addition, the strain gradients in the experiments may numerically differ from the theoretical results, and the primary ratiocination for this unconformity may be that the simulation calculations were performed under the assumption: as the material is homogeneous, isotropic, and perfectly elastic, and superficial friction is negligible and the surface is ideally smooth. However, in actual measurements, whereas defects or impurities may exist on the crystal surface.

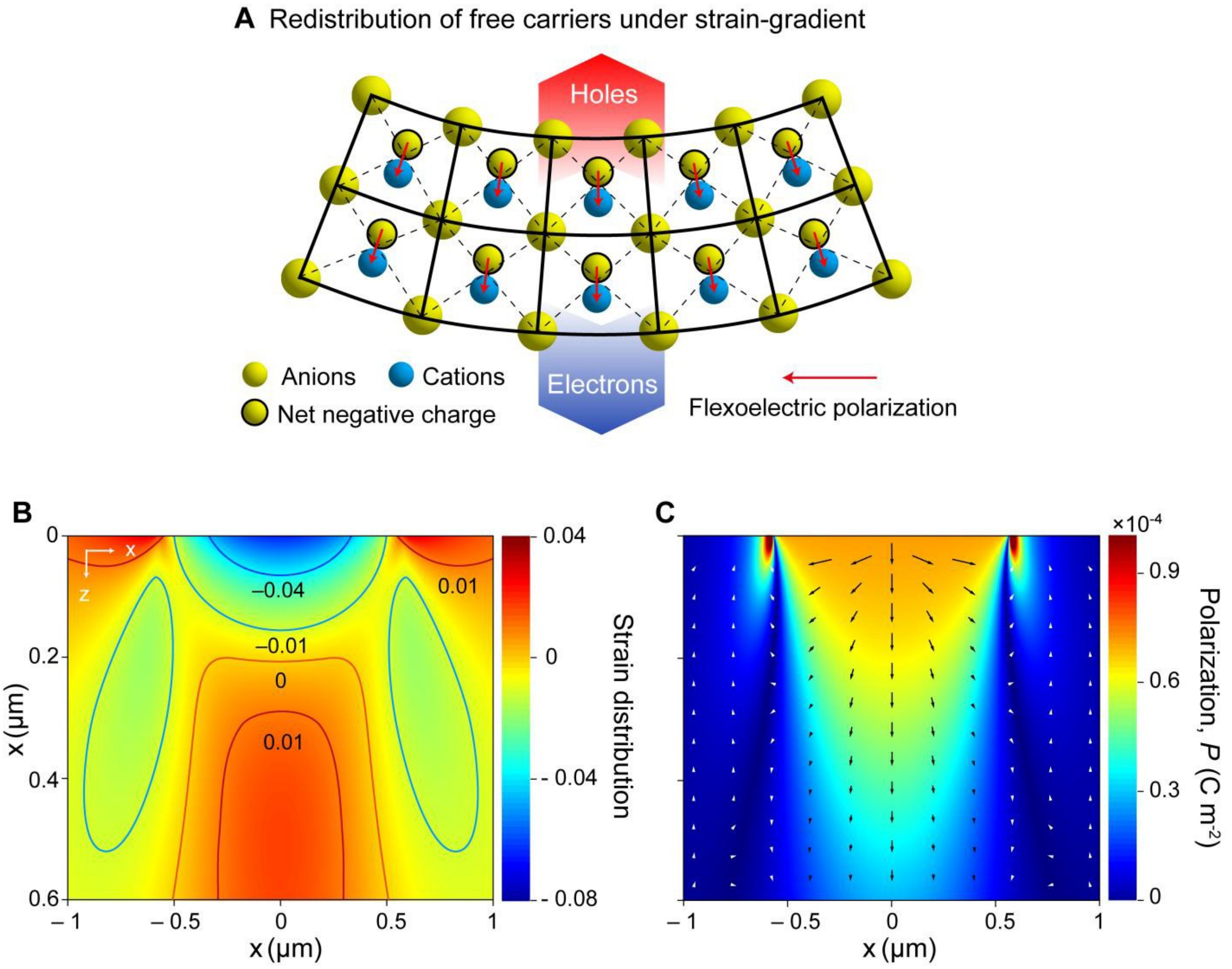

Fig. S1. The mechanism of flexoelectronics. (A) The flexoelectric polarization generated in the inhomogeneously strained crystal, which exerts a significant influence on the concentration and distribution of free carriers at the channel and metal-semiconductor interface. **(B)** Theoretical simulation of strain distribution in Si under indentation of 25 mN with tungsten probe (radius, 1 μm). **(C)** Corresponding flexoelectric polarization distribution in Si.

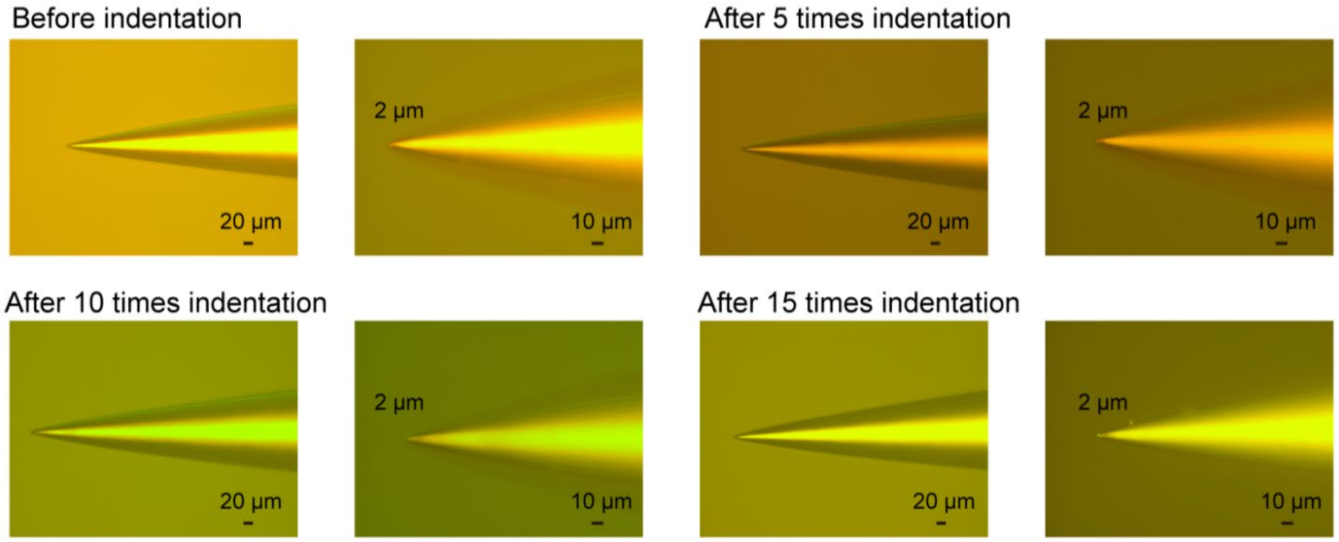

Fig. S2. Optical microscope photographs of tungsten probe before and after multiple indentations.

Supplementary Text 2

Theoretical calculation of flexoelectric polarization distribution in Si

Considering the absence of piezoelectric effects in centrosymmetric semiconductors, the coupling between the mechanical strain gradient and induced electric polarization can be described as(*32*):

$$P_i = \mu_{ijkl} \frac{\partial \varepsilon_{kl}}{\partial x_j} \tag{17}$$

where P_i represents the electric polarization component, μ_{ijkl} represents the flexoelectric coefficient and $\frac{\partial \varepsilon_{kl}}{\partial x_j}$ represents the strain gradient component along the x_j direction. This equation describes the flexoelectric contribution to polarization of the strain gradient, where fourth-order tensor μ_{ijkl} allows it to exist in any material.

For single crystal Si, the non-zero flexoelectric coefficient has three independent components: longitudinal flexoelectric coefficient μ_{1111} (μ_{11}), transverse flexoelectric coefficient μ_{1122} (μ_{12}), and shear flexoelectric coefficient μ_{1212} (μ_{44}). The mathematical expression of μ_{ijkl} can be simplified to:

$$\mu_{ijkl} = \begin{pmatrix} \mu_{11} & \mu_{12} & \mu_{12} & 0 & 0 & 0 \\ \mu_{12} & \mu_{11} & \mu_{12} & 0 & 0 & 0 \\ \mu_{12} & \mu_{12} & \mu_{11} & 0 & 0 & 0 \\ 0 & 0 & 0 & \mu_{44} & 0 & 0 \\ 0 & 0 & 0 & 0 & \mu_{44} & 0 \\ 0 & 0 & 0 & 0 & 0 & \mu_{44} \end{pmatrix} \tag{18}$$

It is worth noting that the flexoelectric coefficients cannot be determined unambiguously owing to the convoluted question of extracting indicidual coefficient components from tests, and there may be orders of magnitude distinctions between theory and experiment(*31, 38, 29*).

The flexoelectric charge density q can be obtained from the aforementioned polarization(*40*).

$$q = -\nabla \cdot P \tag{19}$$

According to the Poisson distribution, the electrostatic properties of the charges are given by:

$$\nabla^2 \psi_i = -\frac{\rho(\vec{r})}{\varepsilon_s} \tag{20}$$

where ψ_i represents the electric potential, $\rho(\vec{r})$ represents the charge density distribution and ε_s represents the dielectric constant of a semiconductor.

The drift and diffusion current-density equations related to electric field, charge density, and local current are given by：

$$\begin{cases} J_n = q\mu_n nE + qD_n\nabla n \\ J_p = q\mu_p pE - qD_p\nabla p \\ J = J_n + J_p \end{cases} \tag{21}$$

where $J_{n(p)}$, $n(p)$, $\mu_{n(p)}$ and $D_{n(p)}$ represent the current density, concentration, mobility and diffusion coefficient of electrons (holes), respectively. And J represents the total current density. Carrier transport driven by an electric field is described by the continuity equation：

$$\begin{cases} \dfrac{\partial n}{\partial t} = G_n - U_n + \dfrac{1}{q}\nabla \cdot J_n \\ \dfrac{\partial p}{\partial t} = G_p - U_p - \dfrac{1}{q}\nabla \cdot J_p \end{cases} \tag{22}$$

where $G_{n(p)}$ and $U_{n(p)}$ represent the generation rate and recombination rate of electrons (holes), respectively. If there is no additional photoexcitation, then G_n=G_p=0. Taking the frequently-used Shockley-Read-Hal recombination as an example, which results in:

$$U_n = U_p = U_{SRH} = \frac{\mathrm{np} - n_i^2}{\tau_p(n+n_1) + \tau_n(p+p_1)} \tag{23}$$

where U_{SRH} represents the Shockley-Read-Hall recombination, $\tau_{n(p)}$ represents the electron (hole) carrier lifetime, n_1 and p_1 represent the trap-level related concentrations of holes and electrons, respectively. And n_i represents the intrinsic carrier concentration.

Therefore, combining the basic semiconductor equations of the electrostatic module [Equation (20)] and the drift-diffusion module [Equation (22)], which reads:

$$\begin{cases} \varepsilon_s \nabla^2 \psi_i = -q(p - n + N + \rho_{flexo}) \\ -\nabla \cdot J_n = -qU_{SRH} \\ -\nabla \cdot J_p = qU_{SRH} \end{cases} \tag{24}$$

where ρ_{flexo} denotes the flexoelectric polarization charges. Applying nonlinear function fitting, the negative polarized charge distribution within the depth x_d along the centerline direction can be described as(*32*)：

$$\rho_{flexo} = \rho_m \left[1 + \frac{x}{x_0} \right]^{-1} \quad x \le x_d \tag{25}$$

where ρ_m represents the peak polarization charges density, x represents the depth, x_0 denotes the decay of the charge distribution.

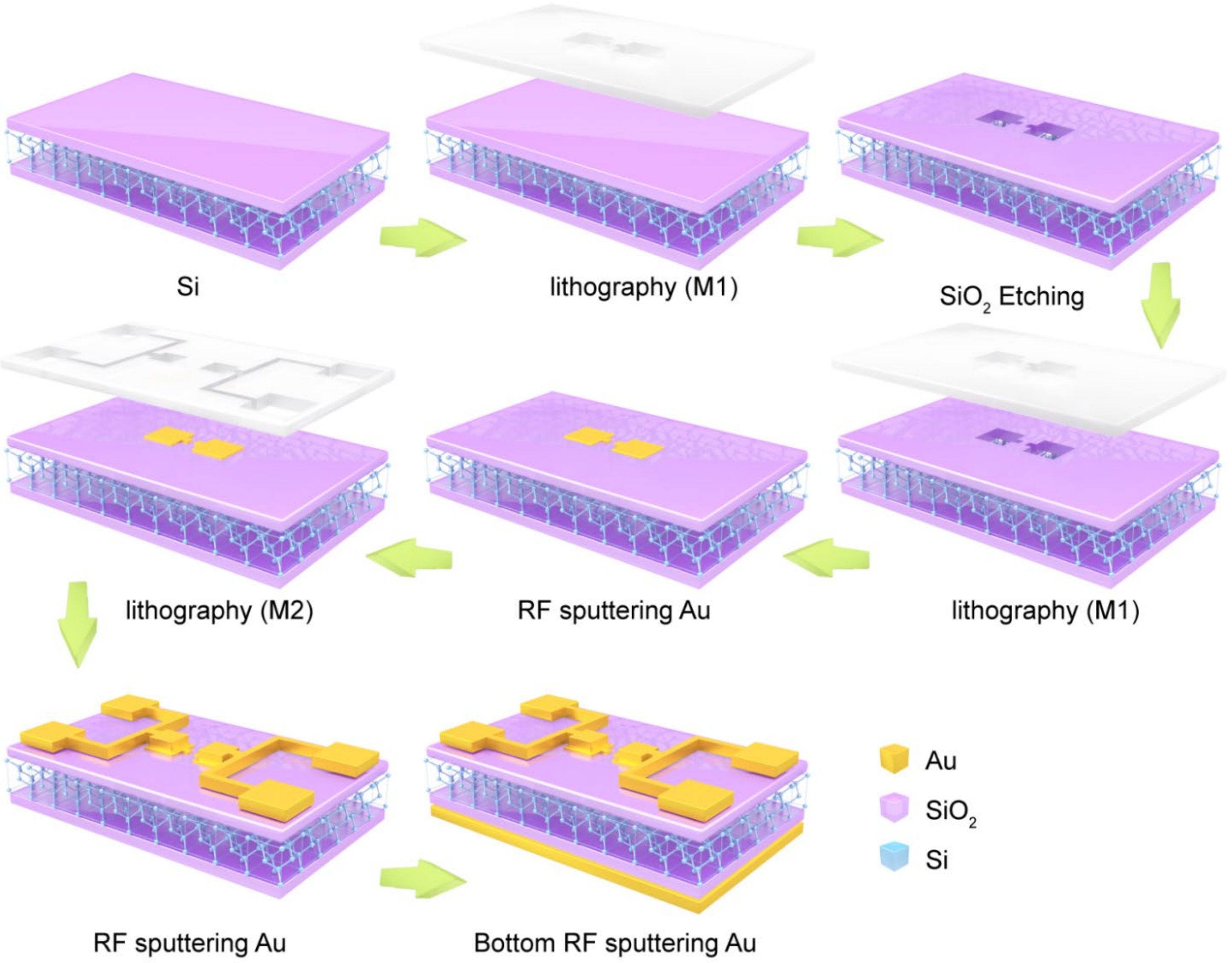

Fig. S3. Fabrication process of the Si field effect transistor.

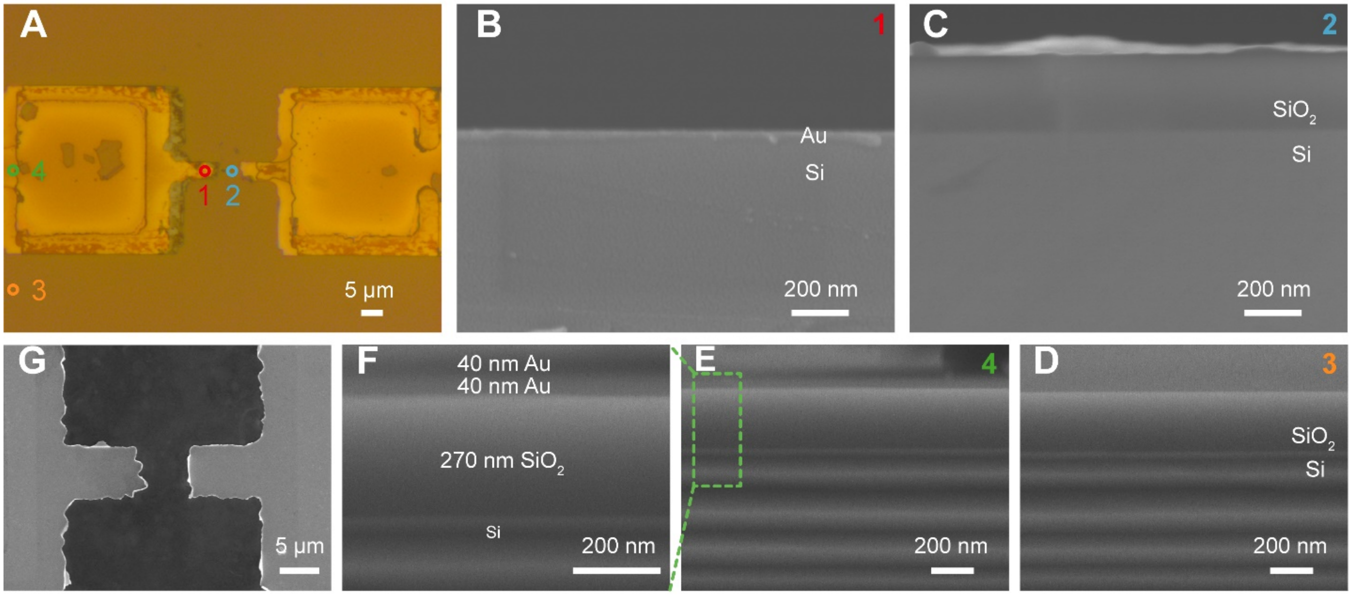

Fig. S4. Characterization of structure and morphology of Si transistor. (**A**) Top-view optical microscope photograph of a Si transistor with 5 um channel width. Cross-sectional SEM images of the heterogeneous stack structure at different locations tagged with red circle (**B**), blue circle (**C**), orange circle (**D**), and green circle (**E**), respectively. (**F**) Enlarged view of the dashed frame shown in E. (**G**) SEM image of the channel of the transistor.

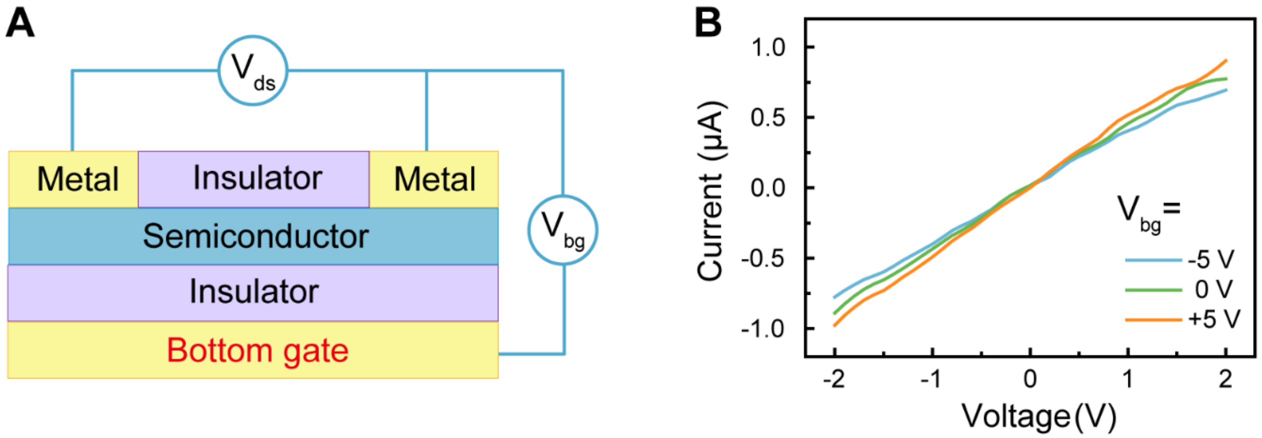

Fig. S5. Electrical characteristics of a Si transistor. (A) Schematic circuit diagram of a Si transistor. **(B)** The corresponding I_{ds}-V_{ds} output characteristics of the transistor.

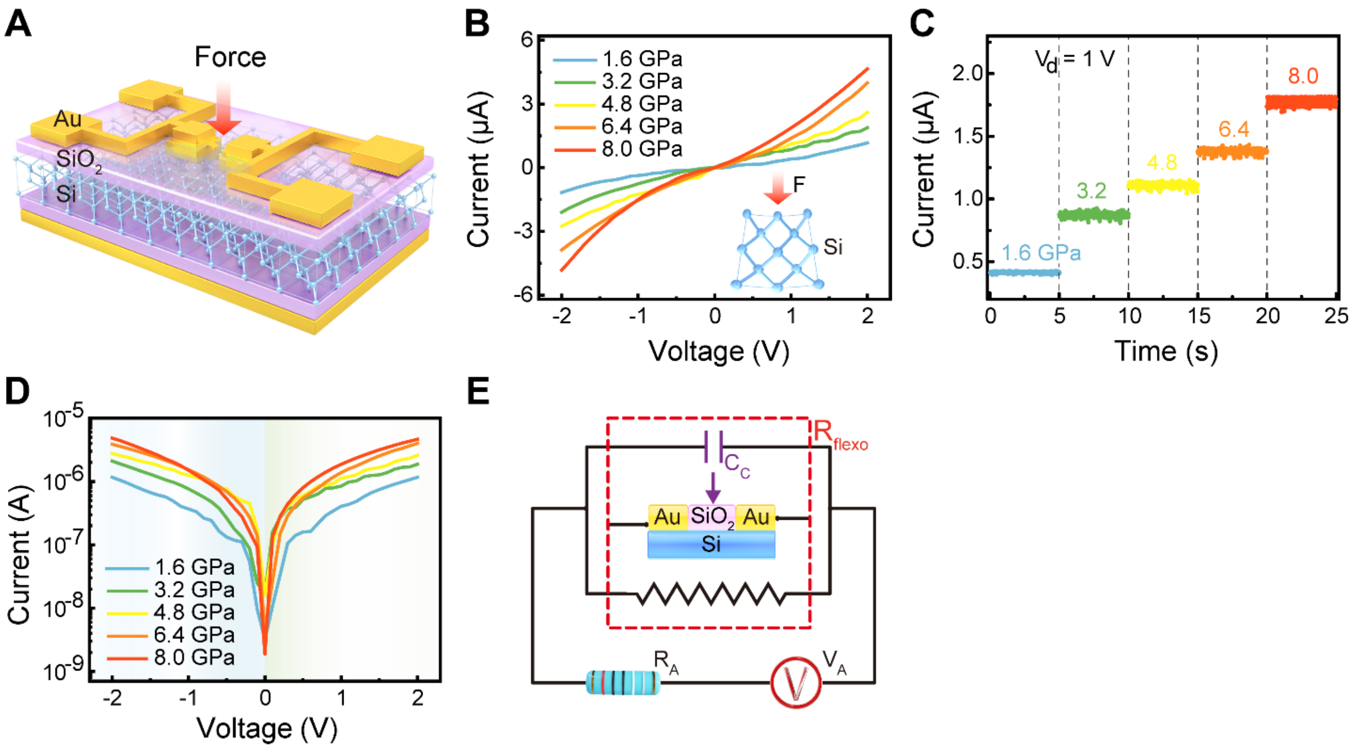

Fig. S6. Reproducible electrical characterization of a Si transistor with indentation on the channel. **(A)** Illustrative schematic of a Si transistor with indentation on the channel. **(B)** The symmetric modulation of electrical transport exhibits the Ohmic characteristic of the channel-width gating effect. **(C)** The current response of SFT increased step-by-step as the loading force increased under a fixed bias of 1 V. **(D)** I-V characteristics in a semi-logarithmic coordinate. **(E)** Equivalent circuit.

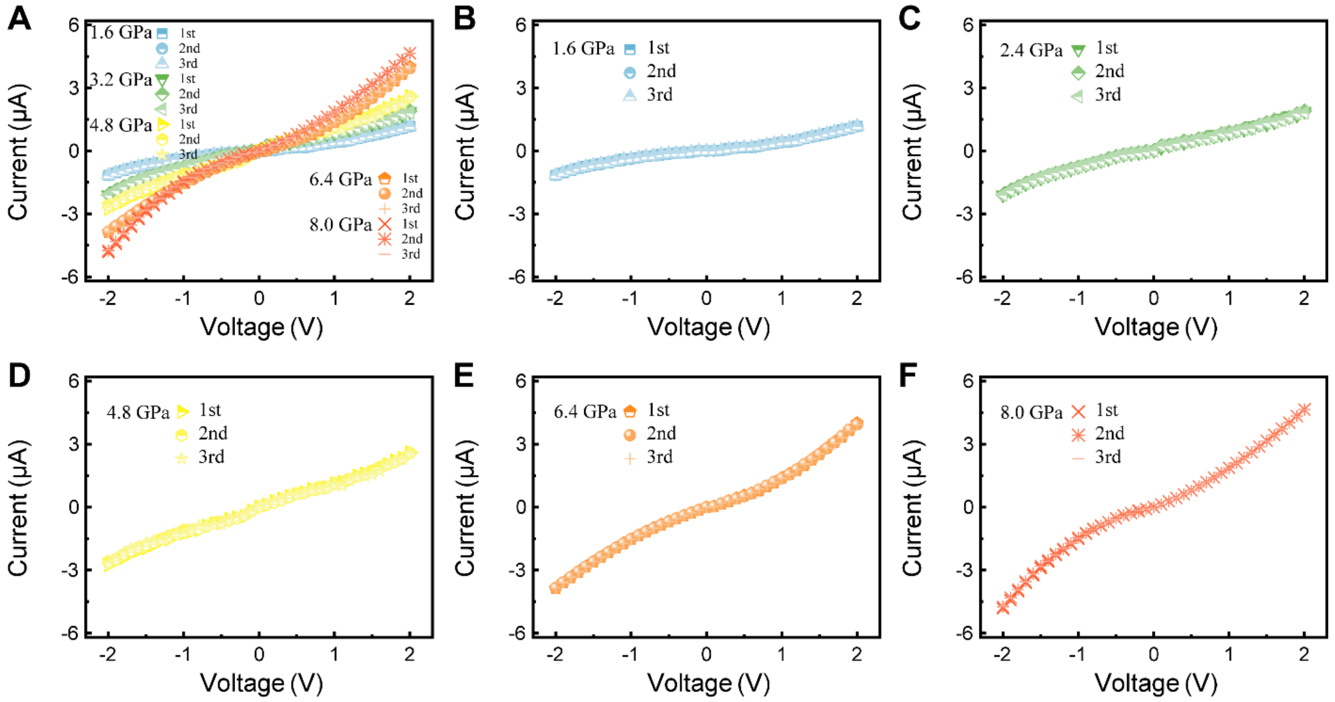

Fig. S7. Repetitive measurement of the I-V characteristics of a Si transistor with indentation on the channel. **(A)** Repeated measurement of transistor under loading forces from 5 mN to 25 mN. **(B)**, **(C)**, **(D)**, **(E)** and **(F)** represent repeated I-V measurement under the loading forces of 5 mN, 10 mN, 15 mN, 20 mN and 25 mN, respectively.

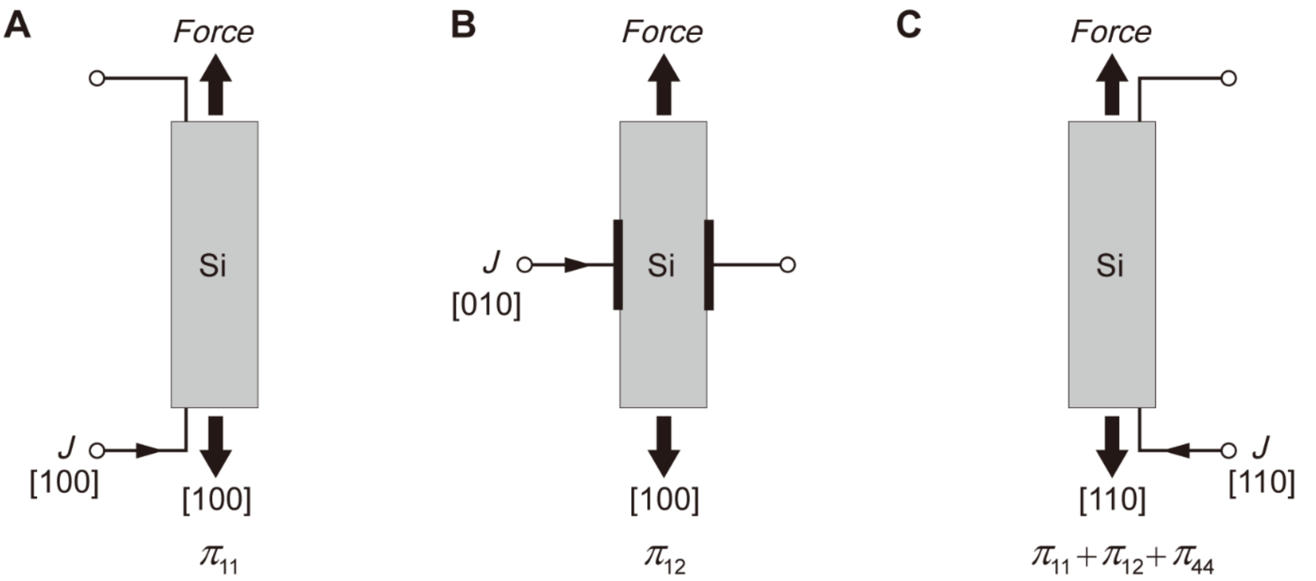

D

Materials	ρ_0 ($\Omega \cdot$cm)	Piezoresistive coefficient (10^{-11} Pa^{-1})					
		π_{11}	π_{12}	π_{44}	$(\pi_{11}+2\pi_{12}+2\pi_{44})/3$	$(\pi_{11}+\pi_{12}+\pi_{44})/2$	$-(\pi_{11}+2\pi_{12})$
p-Si	7.8	6.6	−1.1	138.1	93.5	71.8	−4.4

$$\frac{\Delta\rho}{\rho_0}=\pi F$$

Fig. S8. Piezoresistive effect in bulk p-type Silicon. (A-C) The relationship between the piezoresistive coefficients and the crystal orientation. **(D)** The values of the anisotropic piezoresistive coefficient of p-Si. Piezoresistive effect is a volume effect without polarity, which is the change in resistivity arising from the strain-induced variations in bandgap, charge carrier density and density of states in the semiconductor conduction band.

Supplementary Text 3

Piezoresistive effect in Si

Piezoresistive effect is inevitable when applied a loading force, which results from a change in band gap, charge carrier density or density of states in the conduction band of the strained semiconductor. It is a symmetric volume effect without polarity, which functions as a scalar "resistor". However, the flexoelectronic effect is an interface effect, using the strain-gradient induced flexoelectric polarization to asymmetrically modulate the local contact of the device because of the polarity of the flexoelectric polarization potential, which is similar to the piezotronic effect. The polarity of the flexoelectric polarization depends on that of the applied inhomogeneous stress or strain.

Additionally, the piezoresistive effect in Si exhibits obvious anisotropy, as shown in **fig. S8A-C**. The corresponding piezoresistive coefficient π_{12} of p-type Si in the same configuration with this work is about -1.1×10^{-11} Pa^{-1}, which is a negative value and much small. It cannot explain the anomalous behavior of the channel-width gating of the flexoelectronic transistor. In order to show the influence of piezoresistive effect on the electrical transport, we further investigated the electrical transport properties by applying uniform forces on the channel of Si transistor (**Fig. 2D-F**). The current has an inappreciable variation with gradually increased loading forces, which demonstrate that the role of the piezoresistive effect can be negligible in this case.

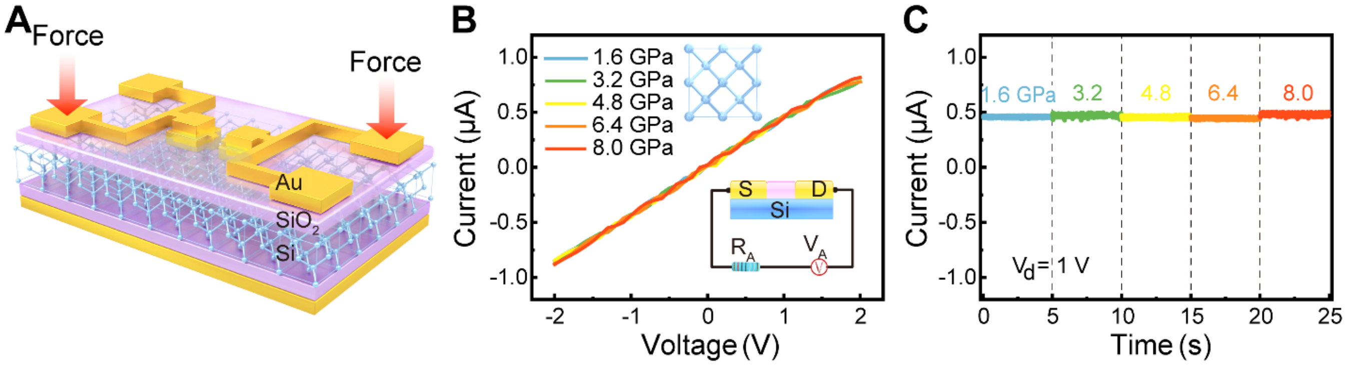

Fig. S9. Electrical characterization of a Si transistor under loading forces. (A) Schematic illustration of a Si transistor under loading forces. **(B)** I-V characteristics of a Si transistor under forces. **(C)** Current outputs of a Si transistor under forces at a fixed voltage of 1 V.

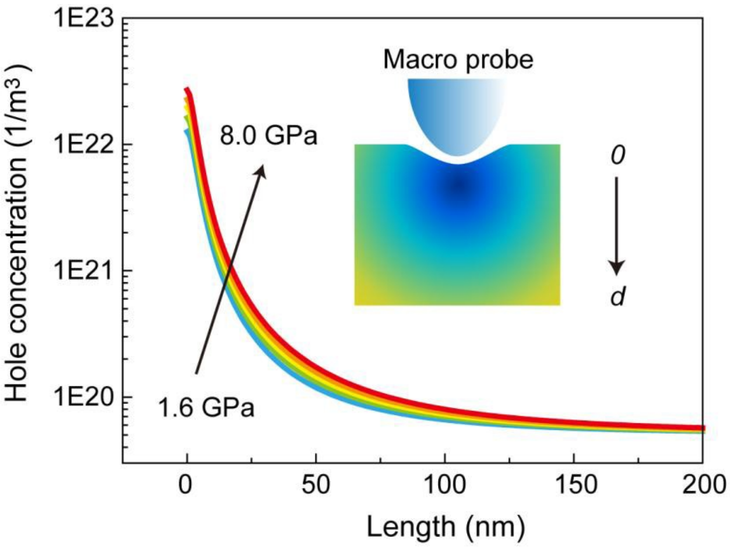

Fig. S10. **Theoretical simulation of flexoelectric polarization in p-Si leads to the change of distribution of holes.**

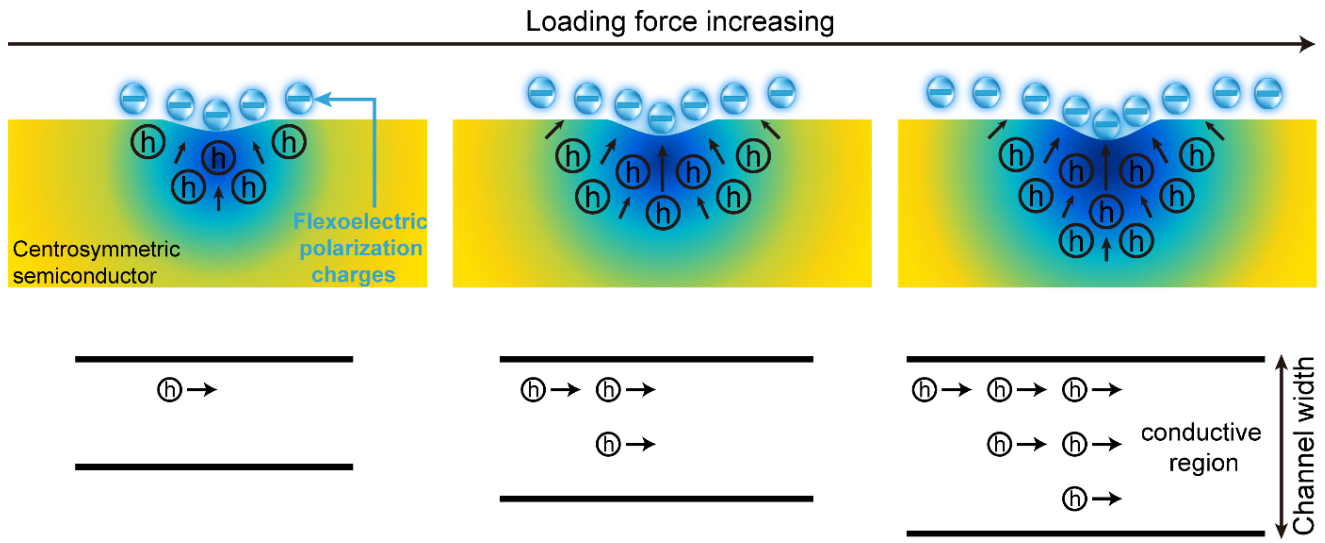

Fig. S11. Schematic illustrating the mechanism of a Si transistor under indentation on the channel. The negative flexoelectric polarization charges attract the holes along the polarization field direction, resulting in the accumulation of holes and the formation of an enhanced region near the top interface of channel.

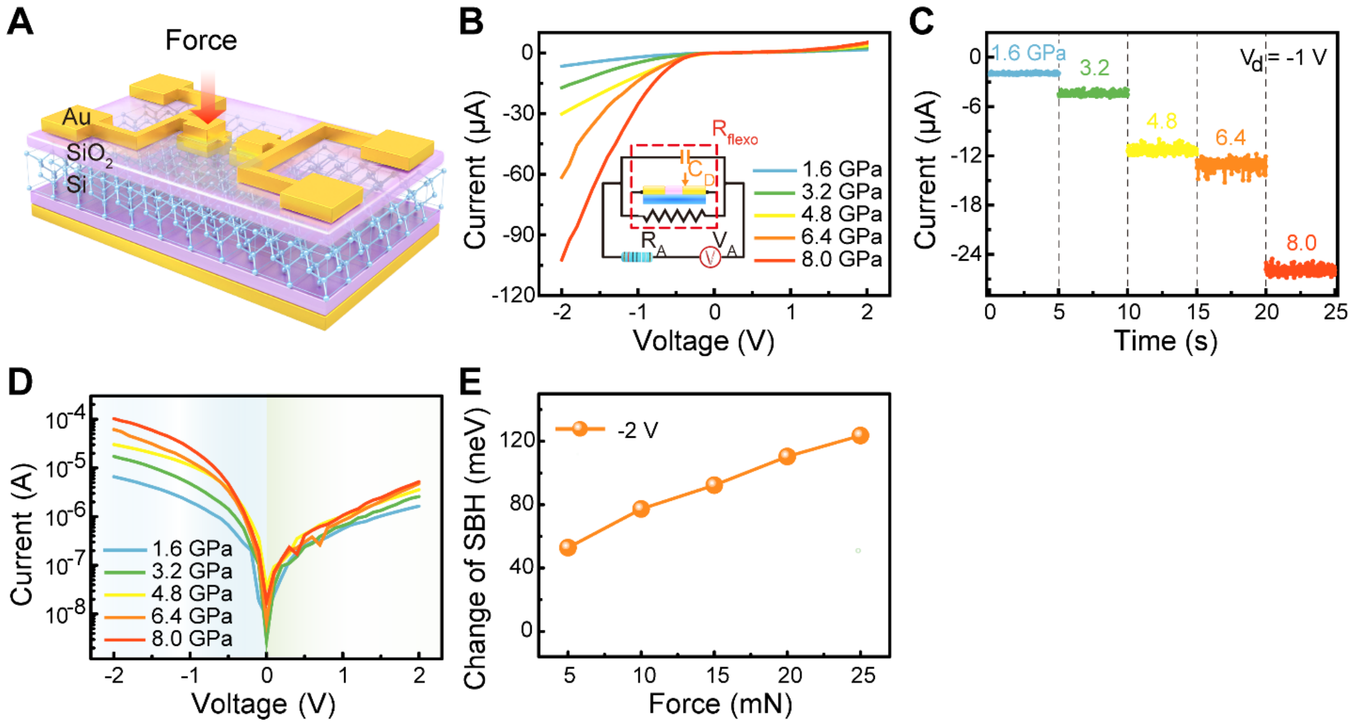

Fig. S12. Reproducible electrical characterization of Si transistor with indentations on the drain. **(A)** Schematic diagram of Si transistor under force on the drain. **(B)** The asymmetric modulation of electrical transport with Schottky characteristic by interfacial barrier gating. **(C)** The current response of SFT increased step-by-step as the loading force increased. **(D)** I-V characteristics in a semi-logarithmic coordinate. **(E)** Variation of SBH derived from the I-V curves of B.

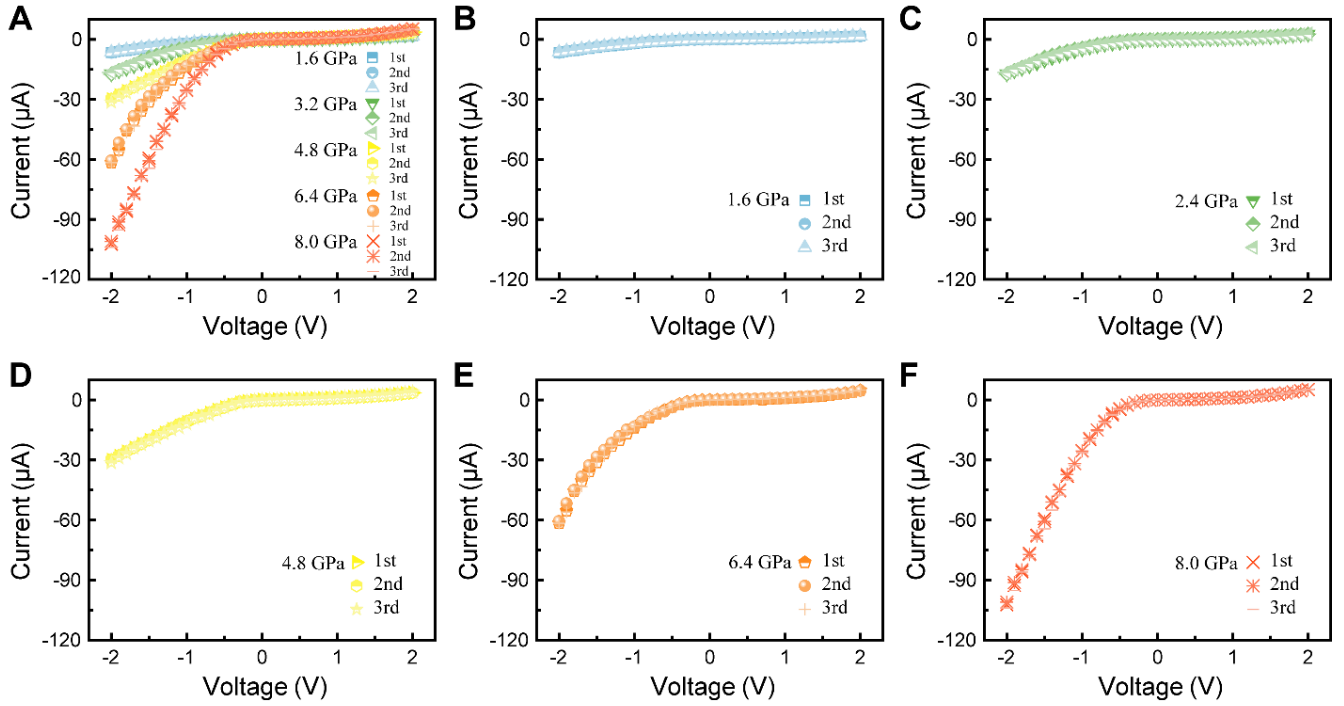

Fig. S13. Repetitive measurement of the I-V characteristics of SFT in interfacial barrier gating. (A) Repeated measurement under loading forces from 5 mN to 25 mN. **(B)**, **(C)**, **(D)**, **(E)** and **(F)** represent repeated I-V measurement under the loading forces of 5 mN, 10 mN, 15 mN, 20 mN and 25 mN, respectively.

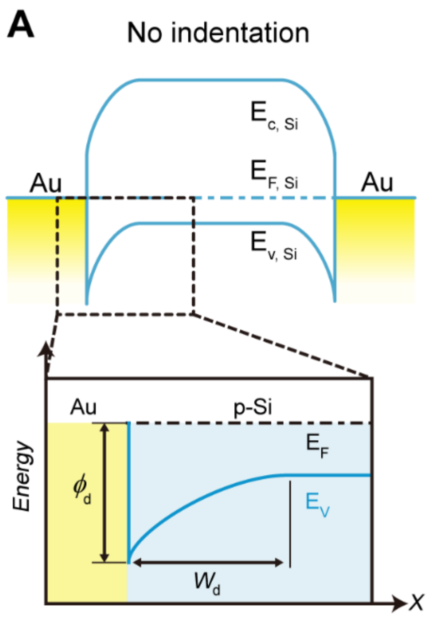

Fig. S14. Band diagrams of SFT in interfacial barrier gating model. The detailed evolution of interfacial barrier height of Au-Si contact under probe indentation. ϕ_d and W_d represent the Schottky barrier height and barrier width of Au-Si contact, respectively.

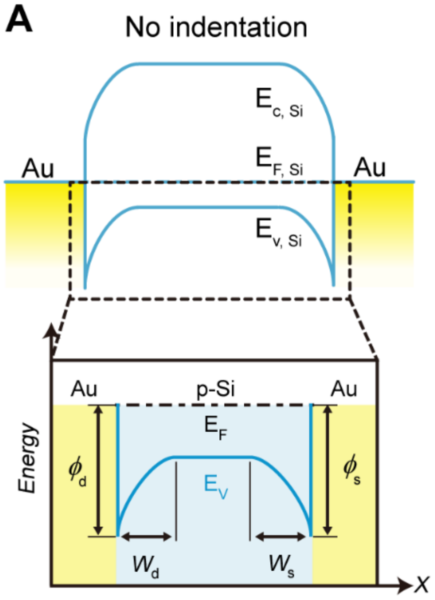

Fig. S15. Band diagrams of SFT under different forces in dual interfacial barrier gating. The detailed evolution of interfacial barriers' height of Au-Si contacts with indentation applied on both of source and drain. ϕ_d, ϕ_s and W_d, W_s represent the Schottky barrier height and barrier width of Au-Si contacts, respectively.

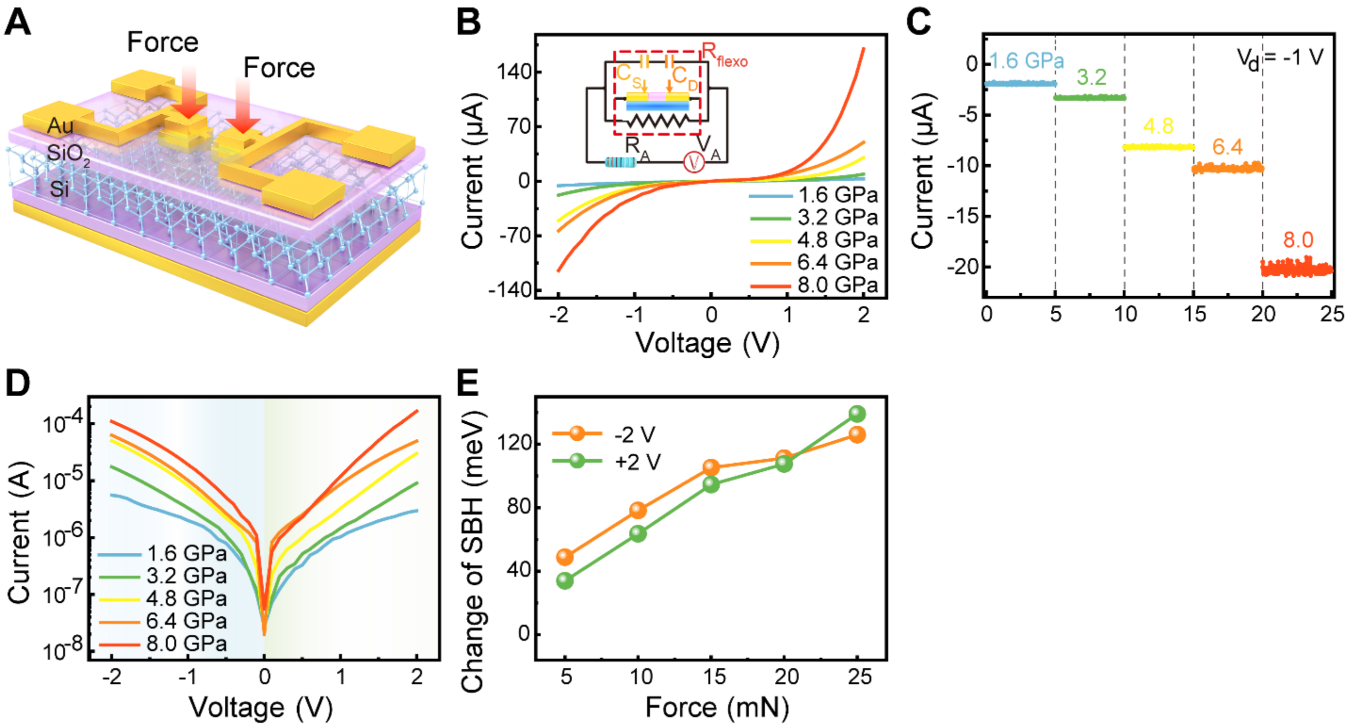

Fig. S16. Reproducible electrical characterization of SFT in dual interfacial barriers' gating. (A) Schematic illustration of the SFT with indentations applied on both of the Au-Si interfaces. **(B)** The symmetric modulation of electrical transport with Schottky characteristic. **(C)** The current response of SFT increased step-by-step as the loading force increased. **(D)** I-V output characteristics in a semi-logarithmic coordinate. (**E**) Variation of SBH derived from I-V curves of B.

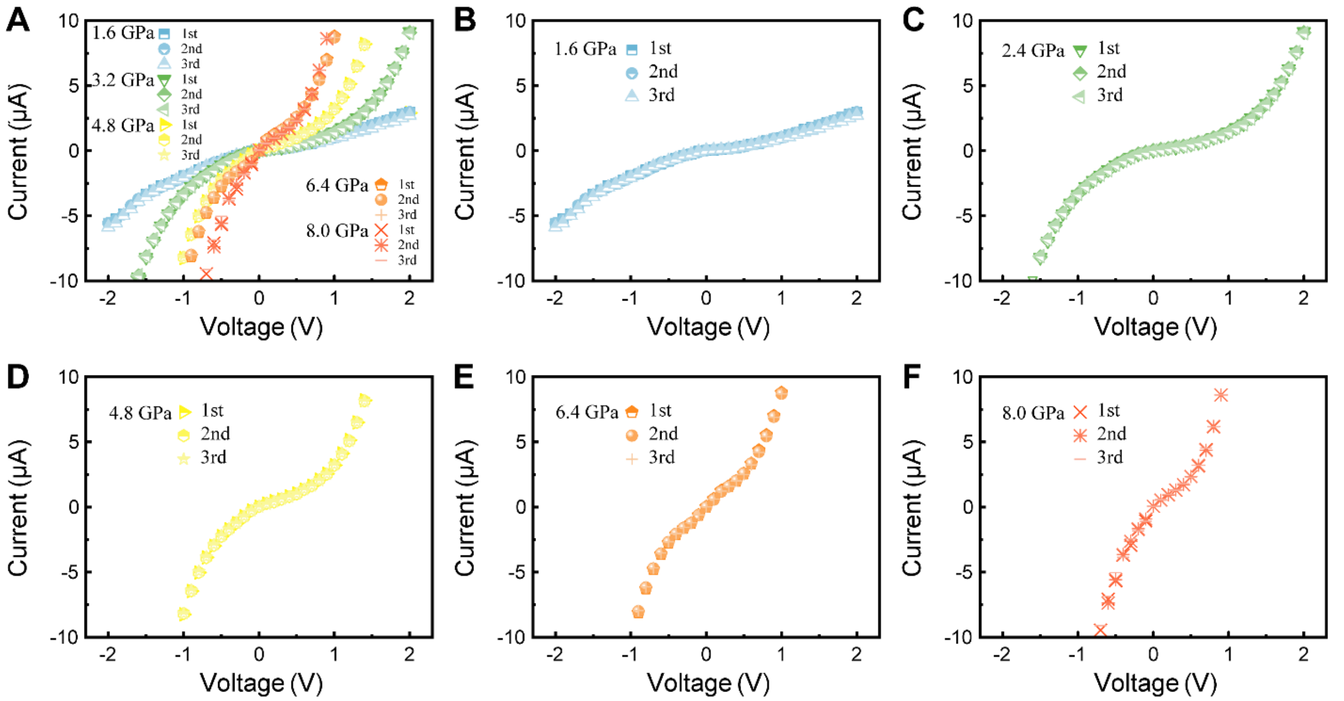

Fig. S17. Repetitive measurements of I-V characteristics of SFT in dual interfacial barriers' gating. (A) Repeated measurement under loading forces from 5 mN to 25 mN. **(B)**, **(C)**, **(D)**, **(E)** and **(F)** represent repeated I-V measurement under the loading forces of 5 mN, 10 mN, 15 mN, 20 mN and 25 mN, respectively.

Supplementary Text 4

Mathematical calculation of the gauge factor of SFT

The sensitivity of the flexoelectronic transistor utilizes gauge factor (*GF*) to quantitatively characterize the performance of the devices, which can be generally calculated by using the ratio of the relative current variation to the mechanical tip-strain, expressed as(*41*):

$$GF = \left[\left(I_{strain} - I_{free} \right) \big/ I_{free} \right] \big/ \Delta\varepsilon \tag{27}$$

$$\sigma = E\varepsilon \tag{28}$$

where I_{strain} represents the current measured under the strain gradient, I_{free} represents the initial current measured under strain free condition, $\Delta\varepsilon$ is the strain.

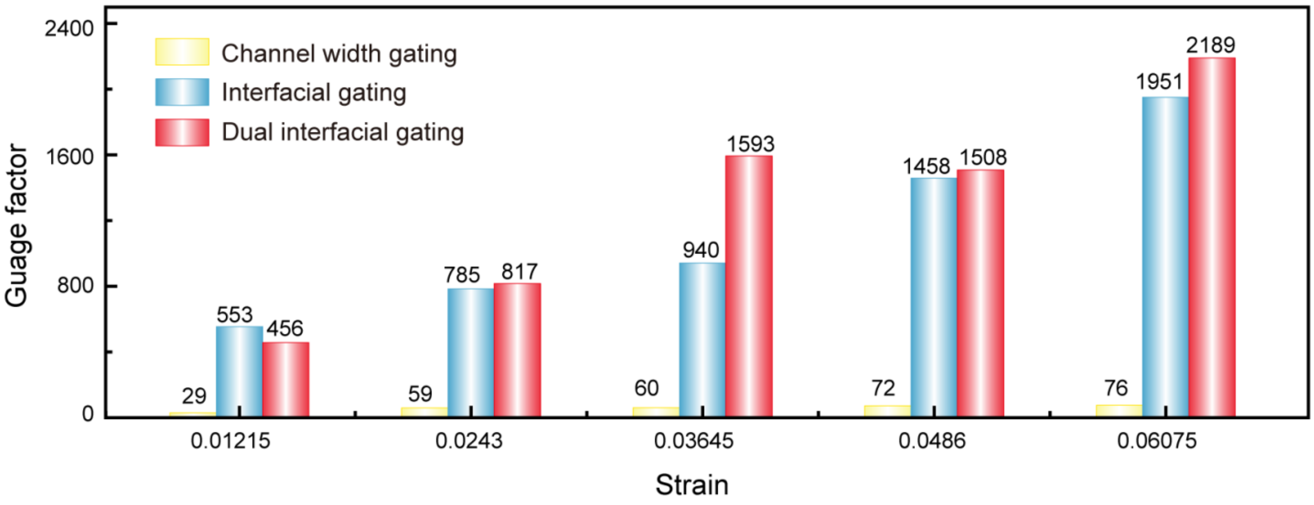

Fig. S18. Gauge factor of SFT in three gating modes.

Table S1. Comparison of Strain Sensing Works

Year	Active materials	Sensing mechanism	Gauge factor	Ref.
	Bulk p-Si (device scale)	Flexoelectronic	2189	This work
2020	p-Si (nano-indentatiaon)	Flexoelectronic	2650	[32]
2020	Bulk TiO_2 single crystal	Flexoelectronic	~472	[32]
2020	Nb-$SrTiO_3$ single crystal	Flexoelectronic	~183	[32]
2021	GaN thin films	Piezotronic	2206	[42]
2019	ZnO nanowire	Piezotronic	~435	[43]
2021	CdS nanospheres	Piezotronic	236	[44]
2019	2D MoS_2 monolayer	Piezotronic	~140	[45]
2015	GaN nanowire	Piezotronic	1126	[46]
2015	ZnO cluster	Piezotronic	784	[47]
2014	ZnO array	Piezotronic	1813	[48]
2014	ZnO bulk	Piezotronic	150~800	[49]
2018	$PtSe_2$	Piezoresistive	85	[50]
2017	Mxene (Ti_3C_2)	Piezoresistive	180.1	[51]
2016	MoS_2 thin layer	Piezoresistive	56.5~72.5	[52]
2014	Bulk MoS_2	Piezoresistive	200	[17]

2016	Graphene membranes	Piezoresistive	6.73	[53]
2015	Nanographene	Piezoresistive	600	[54]
2014	Graphene woven fabric	Piezoresistive	35	[55]
2015	Si whisker	Piezoresistive	~225	[56]
2015	Hybrid SWCNTs	Piezoresistive	62	[57]
2014	Rubber/MWCNTs/rubber	Piezoresistive	43	[58]
2014	AgNWs composite	Piezoresistive	14	[59]
2014	Nanoscale crack junction	Piezoresistive	2000	[60]

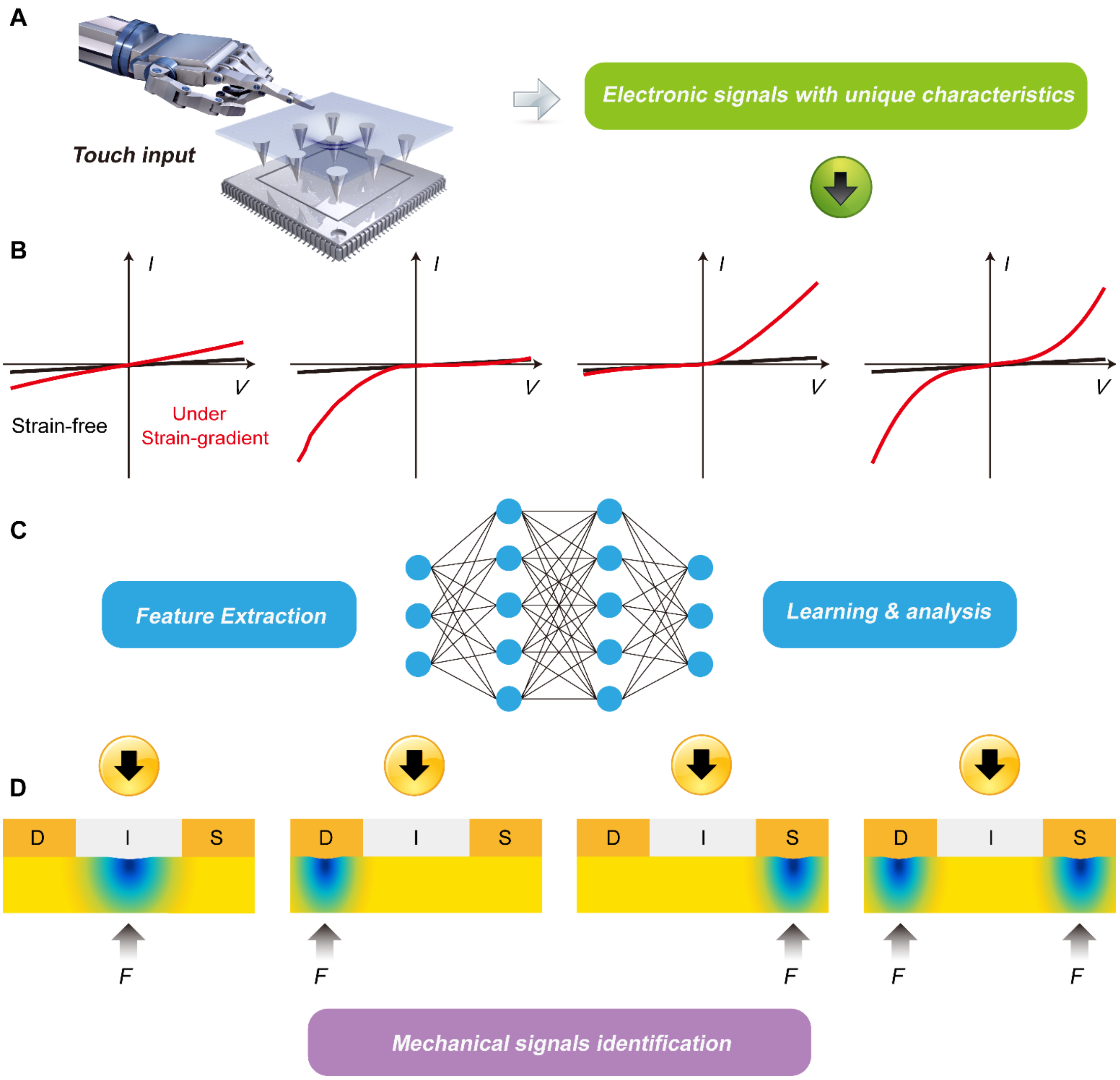

Fig. S19. Prototype of perception system for mechanical signals identification. Mechano-sensory process: **(A)** Mechanical actions interface with perceptual component of the system; **(B)** Generated electronic signals with various characteristics. Identification process: **(C)** Feature of the identified electronic signal captured through deep learning; **(D)** Identification of the mechanical action.

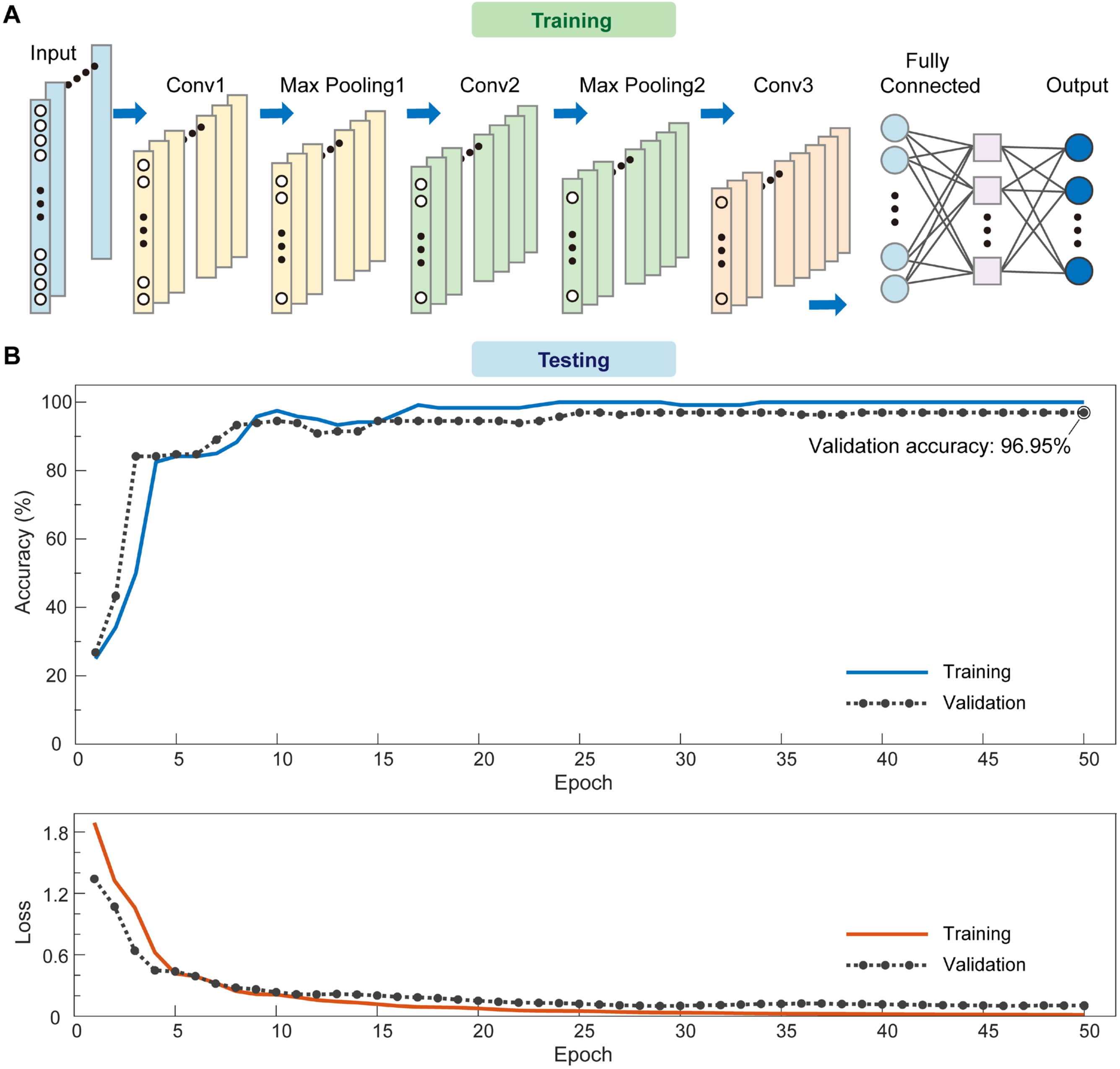

Fig. S20. Mechanical signals identification based on the convolutional neural networks (CNN). (A) Final structure of CNN after optimization. The convolutional layers and max-pooling layers serve as the core modules to automatically implement feature extraction and dimension reduction operations. The features are re-fitted through the fully connected layer, finally using the logistic regression classifier for high-precision identification output in the output layer. **(B)** Recognition accuracy and cross entropy loss of mechanical signals identification with different training epochs, of which the recognition accuracy would gradually reach 96.95% after 50 training epochs.
